# Limited sex differences in plastic responses suggest evolutionary conservatism of thermal reaction norms: A meta‐analysis in insects

**DOI:** 10.1002/evl3.299

**Published:** 2022-11-02

**Authors:** Tiit Teder, Kristiina Taits, Ants Kaasik, Toomas Tammaru

**Affiliations:** ^1^ Department of Zoology, Institute of Ecology and Earth Sciences University of Tartu Tartu EE‐50409 Estonia; ^2^ Department of Ecology, Faculty of Environmental Sciences Czech University of Life Sciences Prague Prague 165 21 Czech Republic

**Keywords:** Body size, development rate, evolutionary constraint, growth rate, sexual bimaturism, sexual size dimorphism, thermal sensitivity, thermal trait

## Abstract

Temperature has a profound effect on the growth and development of ectothermic animals. However, the extent to which ecologically driven selection pressures can adjust thermal plastic responses in growth schedules is not well understood. Comparing temperature‐induced plastic responses between sexes provides a promising but underexploited approach to evaluating the evolvability of thermal reaction norms: males and females share largely the same genes and immature environments but typically experience different ecological selection pressures. We proceed from the idea that substantial sex differences in plastic responses could be interpreted as resulting from sex‐specific life‐history optimization, whereas similarity among the sexes should rather be seen as evidence of an essential role of physiological constraints. In this study, we performed a meta‐analysis of sex‐specific thermal responses in insect development times, using data on 161 species with comprehensive phylogenetic and ecological coverage. As a reference for judging the magnitude of sex specificity in thermal plasticity, we compared the magnitude of sex differences in plastic responses to temperature with those in response to diet. We show that sex‐specific responses of development times to temperature variation are broadly similar. We also found no strong evidence for sex specificity in thermal responses to depend on the magnitude or direction of sex differences in development time. Sex differences in temperature‐induced plastic responses were systematically less pronounced than sex differences in responses induced by variations in larval diet. Our results point to the existence of substantial constraints on the evolvability of thermal reaction norms in insects as the most likely explanation. If confirmed, the low evolvability of thermal response is an essential aspect to consider in predicting evolutionary responses to climate warming.

1 **Impact Summary**

Temperature profoundly affects the growth and development of ectothermic organisms, such as insects. To a considerable extent, phenomena such as the increase in growth rate in response to increasing temperatures are based on fundamental physical principles. Somewhat surprisingly, however, the question of how much is there beyond physics is rarely asked. In other words, we do not know how much and how can ecologically based natural selection modify such responses to temperature. We believe that a systematic approach to this problem can substantially contribute to disentangling the roles of constraint and adaptation in growth schedules, something that is bound to considerably enhance our general understanding of evolutionary processes. As a novel contribution to this field, we use a literature‐derived database to compare thermal responses in development time between male and female insects. Males and females share largely the same genes, but natural selection affects them differently. We therefore interpret similarity between the sexes as evidence of an essential role of physiological constraints, whereas substantial sex differences in responses to varying temperatures are seen as traces of sex‐specific action of natural selection. Our analysis revealed a high degree of similarity between the sexes, thereby supporting the view that constraints play a major role in the evolution of thermal responses in insect growth schedules. We also show that sex differences in thermal plastic responses are considerably less pronounced than sex differences in responses induced by variations in larval diet. The conservatism of thermal responses is a crucial aspect to consider in predicting evolutionary responses to climate warming.

Temperature is a key environmental determinant of growth and development in ectothermic organisms. Within the range of nonstressful temperatures, metabolic rates accelerate with increasing temperatures (Cossins and Bowler 1987; Angilletta 2009), leading to increased growth rates and reduced development times (Atkinson 1994; Kingsolver and Huey 2008). Moreover, in ectothermic animals with determinate growth, individuals developing at higher temperatures tend to terminate their growth at smaller body sizes, a prominent pattern known as the temperature‐size rule (Atkinson 1994; Angilletta and Dunham 2003; Forster et al. 2012; Zuo et al. 2012; Horne et al. 2017). The effects of temperature on ectotherm growth are mirrored in intraspecific latitudinal clines of body size (James's rule; James 1970; Horne et al. 2015) and voltinism (Zeuss et al. 2017; Teder 2020), as well as in shrinking body sizes (Verberk et al. 2021) and shorter generation times (Parmesan 2006; Altermatt 2010) in response to the ongoing climate warming.

Although temperature‐induced plastic responses in animal phenotypes are pervasive, it is not well understood to what extent such physiologically based responses can be adjusted by ecologically driven selection pressures (Svensson et al. 2020). A promising yet underexploited way to evaluate the potential for adaptive adjustments in thermal reaction norms is to compare temperature‐induced plastic responses in males and females (see also Lailvaux 2007; Chelini et al. 2019; Bodensteiner et al. 2020; Blanckenhorn et al. 2021; Pottier et al. 2021). In particular, although conspecific females and males largely share genetic makeup and juvenile environment, selection pressures on adult life histories typically differ between the sexes (Badyaev 2002). The presence of substantial sex differences in plastic responses could thus be interpreted as resulting from sex differences in life‐history optimization. By contrast, low levels of sex‐specific plasticity may (but need not) indicate that certain constraints—physiological, developmental, or genetic—prevent such optimization of the phenotypes to environmental variation.

Females and males indeed often differ in the levels of phenotypic plasticity they express in response to environmental variation. Studies on sex differences in body size plasticity have been especially insightful. In insects in particular, sexes frequently differ in their plastic responses to food limitation, food quality, and larval density, with the larger sex—typically females in insects—displaying a stronger response than the smaller sex (Teder and Tammaru 2005; Stillwell et al. 2010; Rohner et al. 2018). This pattern becomes particularly evident in species with pronounced sexual size dimorphism, that is, when size optima for males and females strongly differ (Teder and Tammaru 2005). Intriguingly, however, plastic size responses to temperature show no consistent sex difference in arthropods, nor are they associated with the direction and magnitude of sexual size dimorphism (Hirst et al. 2015). These contrasting patterns may suggest that the extent to which individuals adaptively adjust their reaction norms shows a systematic dependence on the nature of the environmental factor.

In contrast to body size, sex differences in (thermal) plasticity of other growth‐related traits appear not to have been systematically evaluated. This is unfortunate because studying such relationships is bound to provide new insights into the evolution of adaptive plasticity in growth schedules (Gotthard and Nylin 1995; Pigliucci 2001; Ghalambor et al. 2007; Chelini et al. 2019; Blanckenhorn et al. 2021) and contribute to the question of evolvability of thermal sensitivity in general (Angilletta 2009). Sex differences in development time plasticity deserve particular attention, as development time is a trait closely related to fitness in a wide variety of organisms. In particular, longer development confers a higher risk of succumbing to natural enemy attack and dying without leaving any progeny (Stearns 1992; Blanckenhorn 2000). Development time effects on fitness may also result from the trade‐off between development time and body size: larger individuals are generally more fecund within species (Honek 1993), whereas—everything else being equal—attaining a larger size requires a longer (and riskier) juvenile development (Blanckenhorn 2000; Tammaru et al. 2010; Teder et al. 2021). Therefore, knowledge of sex‐specific responses in development time can also shed further light on environmentally induced variation in sexual size dimorphism (Teder and Tammaru 2005; Hirst et al. 2015). Quite obviously, understanding the limits of (sex‐specific) thermal plasticity is also crucial for predicting species’ responses to climate warming, such as phenological changes and range shifts (Seebacher et al. 2015; Sgro et al. 2016; Pottier et al. 2021).

In this study, we examine sex differences in phenotypic plasticity by performing a meta‐analysis of sex‐specific thermal responses in insect development times. For this purpose, we take advantage of a large number of experimental case studies, conducted in various contexts, which report male and female development times for multiple subsets of individuals reared under different temperature treatments. Data on 161 species with a wide phylogenetic and ecological coverage allow us to make broad generalizations about sex differences in thermal plasticity of development duration across the class of insects. We proceed from the assumption that thermal plasticity in development time is primarily physiologically mediated (constraint‐based) if males and females do not differ in their thermal plastic responses (the null hypothesis, see *Discussion* for justification). By contrast, systematic sex differences in responses to temperature would indicate that thermal reaction norms readily respond to ecologically based selective pressures. As a particular prediction of this differential plasticity hypothesis, we expect the sex with a longer development time to be more responsive to temperature (cf. Rohner et al. 2018). Finally, we compare the magnitude of sex differences in thermal plastic responses with those in response to variation in diet (recently reviewed by Teder et al. 2021). Sex‐specific diet‐induced responses provide a useful reference point for judging the magnitude of sex differences in thermal plasticity. We further integrate the revealed patterns with previously published data on plastic responses in body size (Teder and Tammaru 2005; Stillwell et al. 2010; Hirst et al. 2015). This allows us to examine the bivariate thermal reaction norms for size and time at maturity and compare them to those describing diet‐induced reaction norms of these traits. We discuss the results in light of constraints on the evolvability of insect developmental schedules.

## Material and Methods

### DATA SEARCH

To evaluate sex differences in thermal plasticity in development times, we made use of experimental studies reporting sex‐specific data on insect development times in conspecifics reared under two or more temperature treatments. The majority of primary papers for the present study were collected by the lead author (T. Teder) as a result of a systematic screening of major journals in the field of entomology, ecology, and evolutionary biology over the period of almost two decades (since 2004). Another significant proportion of data was derived from the databases that we had collated for our previous synthetic works on insect life histories (Teder and Tammaru 2005; Blanckenhorn et al. 2007; Teder et al. 2008; Stillwell et al. 2010; Molleman et al. 2011; Teder 2014; Rohner et al. 2018). The bulk of primary data for these earlier studies had been identified by a retrospective screening of relevant journals (including volumes from the period from the 1970s to the early 2000s). In both cases, our systematic screening procedure included full‐text reviewing of articles identified as “promising” on the basis of titles in the tables of contents of the journals’ volumes. As the desired data are typically reported in tables and figures, locating relevant data within the articles was straightforward.

Additional studies were identified by a thorough search in major literature databases (Google Scholar, Web of Science). Importantly, search queries used to find relevant data were neutral with regard to the main questions of our study (i.e., sex differences in thermal plasticity). In particular search queries, we employed combinations of three search terms: “temperature,” one of several insect orders (or “insects”), and one of several synonyms of development time (developmental/development/egg‐to‐adult/larval/nymphal time/period/duration). For a subset of species, data collected for a recent meta‐analysis (Teder et al. 2021) allowed us to derive comparable estimates of sex difference in diet‐induced plasticity (see below for details).

We strongly believe that the chosen approaches to data collection were unlikely to produce any biases that might have affected the findings and conclusions of the present study, not least because a vast majority of the primary studies addressed questions not interfering with the objectives of the present analysis.

### DATA EXTRACTION AND CRITERIA FOR INCLUSION

From each source paper, we extracted data on average total and/or larval development time separately for males and females for as many temperature treatments (between 2 and 11, average 4; Table S1) as reported. These data were treated as primary datasets. The time from oviposition to adult emergence was the preferred measure of total development time, but the time from egg hatching to adult emergence was also accepted. Larval development time was typically measured as the time from egg hatching (or sometimes oviposition) to pupation in holometabolous species and from egg hatching (or oviposition) to adult emergence in hemimetabolous species.

We limited examining sex differences in thermal plasticity to manipulative experiments in which conspecific individuals were reared under different (mostly constant) temperature treatments in otherwise uniform conditions. Measurements extracted from different source studies were always treated as separate primary datasets. However, data from a single study could also be split into multiple primary datasets if obtained from different experiments or using different species/populations/genotypes. Data from multifactorial experiments were divided into multiple datasets so that temperature could vary while other environmental factors (e.g., food quality/quantity, larval density) were kept constant. WebPlotDigitizer (A. Rohatgi; https://automeris.io/WebPlotDigitizer) was used to extract graphically presented data.

### OPERATIONS WITH PRIMARY DATA

To obtain a metric for comparing the thermal sensitivity of male and female development times, we calculated the reduced major axis (RMA) regression slope of male development times on female development times for each primary dataset. The RMA regression slope is a widely used quantitative measure to compare sex‐specific plastic responses of various traits across environments (e.g., Fairbairn 1997; Hirst et al. 2015; Rohner and Blanckenhorn 2018; Teder et al. 2021). The paired measurements of male and female average development times from each temperature treatment entered these regression models as individual data points. As we were interested in the relative responses of the two sexes, the variables on the axes (i.e., male and female development times) were logarithmically transformed before slope calculations. These regression slopes were themselves logarithmically transformed (hereafter ln‐slope) to scale their values to a distribution symmetric around zero. In the meta‐analytic procedures (see below), the ln‐slopes were used as effect sizes quantifying sex differences in development time plasticity in source studies. An ln‐slope of zero corresponds to an equal relative change with temperature in the two sexes. In that case, the relative difference in female and male development times does not change with increasing development time. By contrast, significant deviations from an ln‐slope of zero imply that either female (ln‐slope < 0) or male (ln‐slope > 0) development time is thermally more sensitive.

For each ln‐slope, we derived sampling variance employing an approach that accounted for two sources of variation: stochastic among‐treatment variance (derived from the number and concordance of temperature treatments within a study) and within‐treatment variance (reflecting the precision of treatment‐specific mean estimates of male and female development times) (see also Teder et al. 2021). Specifically, we applied the following bootstrap‐based procedure to each primary dataset. First, we constructed a uniform distribution around each treatment‐specific mean value, using its standard error to determine the width of the distribution. A random point was then drawn from each such distribution, providing a simulated sample with its sample size equal to the number of treatments in the respective dataset. The obtained sample was used to calculate a simulated ln‐slope. The procedure was repeated 1000 times, and the standard deviation of these 1000 simulated ln‐slopes was used as a variability estimate for the ln‐slope based on reported mean values (see above). The squared inverse of this variability estimate was used to weight individual ln‐slopes in the meta‐analysis (Koricheva et al. 2013). Ln‐slopes derived from studies that reported treatment‐specific means of development times without variability estimates (such as standard deviation with sample size or standard error) could not be included in the meta‐analytic models. However, we considered these slopes in some qualitative assessments of sex‐specific plastic responses.

Additionally, we calculated the mean sex difference in development time for each primary dataset (= sexual development time dimorphism [SDTD]). Depending on data availability, we computed this variable separately for larval (SDTD_larval_) and total development time (SDTD_total_). First, we quantified SDTD for each treatment as SDTD = [(development time of the sex with longer development) / (development time of the sex with shorter development)] – 1. The values of SDTD were arbitrarily assigned a negative sign if the males, and a positive sign if the females, had a longer development time. These treatment‐specific values of SDTD were then averaged for each primary dataset. The resulting values of SDTD are centered around zero, with SDTD = 0 indicating no sex difference in development time. Quantifying sex differences in development time in this way closely follows the practice widely used to express sexual size dimorphism, proposed originally by Lovich and Gibbons (1992).

### META‐ANALYTIC PROCEDURES

First, we asked whether, in general, the temperature‐induced plastic responses in development time tend to be stronger in males or females. For this purpose, we employed a multilevel mixed‐effects meta‐analytic model (Koricheva et al. 2013). Technically, we ran the meta‐analytic model without any moderator variables to pool individual effect sizes (i.e., ln‐slopes of male development times on female development times), and test if the overall mean effect size (i.e., mean ln‐slope) differs from zero across species. In this model, a phylogenetic correlation matrix was used to account for the phylogenetic relationships of the taxa considered (see Appendix S1). The phylogenetic correlation matrix was derived from the Open Tree of Life database (OpenTree et al. 2022) using the R package *rotl* (Michonneau et al. 2016). For most of the target species, phylogenetic information could be directly retrieved from the Open Tree of Life, whereas in a few cases, we relied on data for congeneric species; two species were replaced with noncongeneric species from the same tribe. We also added a study‐level random effect to the model to account for the hierarchical structure of the data and an observation‐level random effect to estimate the residual variance and enable proper partitioning of the variance (Nakagawa et al. 2017).

To assess the significance of the obtained *z*‐statistic, we generated a permutation‐based distribution for reference. To produce a reference distribution for the test‐statistic, male and female development time (together with corresponding standard error values) were randomly permuted within each treatment of each primary dataset. Such an approach was taken, as due to the nature of our slope variability estimates (see OPERATIONS WITH PRIMARY DATA), the distribution of the test‐statistic under the null hypothesis could not be assumed to be Gaussian, and its use as a reference distribution would not have been appropriate. Similar to the interpretation of individual effect sizes (see OPERATIONS WITH PRIMARY DATA), a negative mean effect size was interpreted as indicating a stronger plastic response in females, and a significantly positive mean effect size a stronger response in males.

Apart from evaluating which sex is more plastic in development time across species, we tested the effect of SDTD on sex‐specific thermal plasticity. Accordingly, mean SDTD entered the meta‐analytic model as an independent variable. Phylogenetic relationships of the considered taxa were accounted for as described above, with study‐ and observation‐level random effects similarly included. Again, the significance of the test‐statistic was assessed using a permutation‐based distribution. To produce a reference distribution for the test‐statistic, moderator values were randomly permuted between treatments. We interpreted no effect of SDTD on sex differences in thermal plasticity as indicating strong physiological constraints on adaptive plasticity. By contrast, greater plasticity in the sex with longer development was considered to indicate sex‐related adaptive adjustments in plastic responses. Indeed, it is reasonable to expect that, if adaptive adjustments are possible, the sex requiring more time to obtain its optimum should be more responsive to its thermal environment (cf. Teder and Tammaru 2005; Rohner et al. 2018).

To assess the magnitude of sex differences in thermal plasticity against an appropriate background, we used sex‐specific responses to variations in diet quality/quantity as a reference. In other words, we evaluated whether reaction norms of female and male development times diverge more readily in response to temperature or diet. Here, we used a subset of species for which data on both temperature‐ and diet‐induced plasticity were available. To compare the magnitude of temperature‐ and diet‐induced sex differences in development time plasticity, we contrasted respective standardized ln‐slopes in a pairwise manner. For this purpose, we applied an original permutation test‐based meta‐analysis (see Appendix S2 for a detailed description of the methodology). Such an approach was chosen because the levels of variation in development time caused by thermal and diet gradients strongly differed (on average, there was a 3.27‐ and 1.28‐fold difference between maximum and minimum trait values in primary datasets, respectively), which might have biased the results of a parametric analysis. In this analysis, we used the absolute values of the standardized ln‐slopes, thus placing their values on a scale that disregards which of the two sexes was more plastic. We used data on both larval and total development time in this analysis; however, this was done under the restriction that datasets derived from temperature/diet manipulations for particular species had to be based on the same metric of development time, that is, either larval or total development time.

All meta‐analytic procedures of this study were performed in R version 4.2.0 (R Core Team 2022) using the *metafor* package version 3.4.0 (Viechtbauer 2010).

## Results

### DATABASE

We identified 161 papers reporting sex‐specific development times of insects along a temperature gradient. These papers provided 199 primary datasets on total development times and 158 datasets on larval development times. Of these, 161 and 122 datasets, respectively, had all the necessary information for their inclusion in meta‐analytic procedures. The final database contained data on 161 insect species, spanning 64 families in 11 orders, with Lepidoptera, Hymenoptera, Diptera, and Hemiptera best represented (see Table S1 for sources of primary data).

Both total and larval development times were typically longer in females than in males (138 of 199 datasets and 123 of 158 datasets, respectively; Table S2). However, note that both measures of sex difference in development time, that is, SDTD_larval_ and SDTD_total_, showed substantial qualitative and quantitative variation within clades (Table S1). In all datasets, the duration of development shortened with increasing temperature both in males and females.

### SEX DIFFERENCES IN THERMAL PLASTICITY OF TOTAL DEVELOPMENT TIME

**Figure 1:**
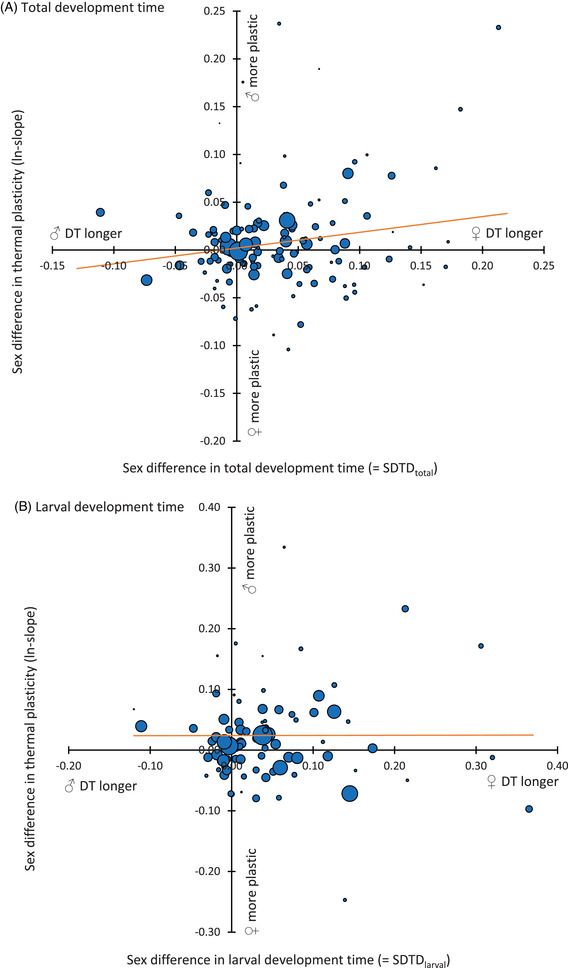
The relationship between sex difference in thermal sensitivity (expressed as ln‐transformed RMA regression slopes of male development time on female development time; referred to as ln‐slopes in the text) and sexual development time dimorphism (SDTD): (A) total development time and (B) larval development time. DT = development time. The trend lines are based on parameters obtained from respective meta‐analytic models. A trend line with a (marginally nonsignificant) positive slope in panel A indicates that thermal sensitivity in total development time tends to be larger in the sex with shorter development time. A flat trend line in panel B indicates that sex differences in thermal plasticity of larval development time do not depend on the direction and magnitude of sex differences in larval development time. Sizes of individual points are proportional to the inverse of error estimates of the ln‐slopes.

There was no overall sex difference in thermal plasticity of total development time across species (mean effect size = 0.013; *P* = 0.12). In this model, phylogenetic structure explained 6% of the variance. We also did not find a significant sex difference for any insect order analyzed separately (Table 1). SDTD_total_ had a marginally non‐significant moderating effect on sex difference in thermal plasticity of total development time (effect size estimate = 0.165, *P* = 0.064). However, contrary to our predictions, sex differences in thermal plasticity tended to decrease with increasing SDTD_total_ (Fig. 1A). This association was primarily driven by species with strongly female‐biased SDTD_total_ in which males tended to display a stronger thermal response than females.

**Table 1:** Meta‐analytically pooled effect sizes (= logarithmically transformed RMA regression slopes of male development time on female development time; referred to as ln‐slopes in the text) calculated to compare sex differences in temperature‐induced plastic responses, separately for total and larval development time

	Total Development Time		Larval Development Time	
Insect Order	Mean Effect Size	*P*‐value	Mean Effect Size	*P*‐value
Lepidoptera	0.007 (19)	0.42	–0.003 (46)	0.85
Coleoptera	–0.003 (20)	0.72	–	–
Diptera	0.007 (20)	0.68	0.090 (21)	0.008^1^
Hymenoptera	0.006 (53)	0.51	–0.013 (10)	0.83
Thysanoptera	–0.029 (5)	0.11	–	–
Hemiptera	0.006 (30)	0.66	–0.012 (30)	0.45
Orthoptera	0.108 (8)	0.06	0.108 (8)	0.06
All species	0.013 (161)	0.12	0.024 (123)	0.24

Negative and positive mean effect size estimates refer to higher thermal sensitivity of females and males, respectively. Corresponding *P*‐values and numbers of primary datasets examined (in parentheses) are shown.

^1^ Not significant after a table‐wide correction for multiple testing.

One cannot a priori exclude that, in some cases, sex differences in plasticity can only be expressed at some part of the temperature gradient. However, as indicated by the nearly invariably strong correlations between male and female development times within source datasets, this is unlikely to be a common phenomenon in insects. Indeed, in as many as 159 of 168 datasets (where data were reported for at least three thermal treatments), the correlation coefficients between male and female development times across temperature treatments were 0.98 or higher (Table S1). This strongly suggests that substantial departures from linearity in the relationship between the responses of males and females are very rare.

### SEX DIFFERENCES IN THERMAL PLASTICITY OF LARVAL DEVELOPMENT TIME

We found no sex difference in thermal plasticity of larval development time (mean effect size = 0.024; *P* = 0.24). In this model, phylogenetic structure explained 11% of the variance. The only significant (but not after a correction for multiple testing) sex difference in thermal plasticity was found separately for Diptera (Table 1). There was no effect of SDTD_larval_ on sex difference in the plasticity of larval development time (effect size estimate = 0.002, *P* = 0.60; Fig. 1B). Again, the correlations between male and female development times across temperature treatments were notably strong in an overwhelming majority of datasets (only studies with at least three thermal treatments considered; Table S1).

### COMPARISON OF SEX DIFFERENCES IN DEVELOPMENT TIME PLASTICITY INDUCED BY TEMPERATURE AND DIET

**Figure 2:**
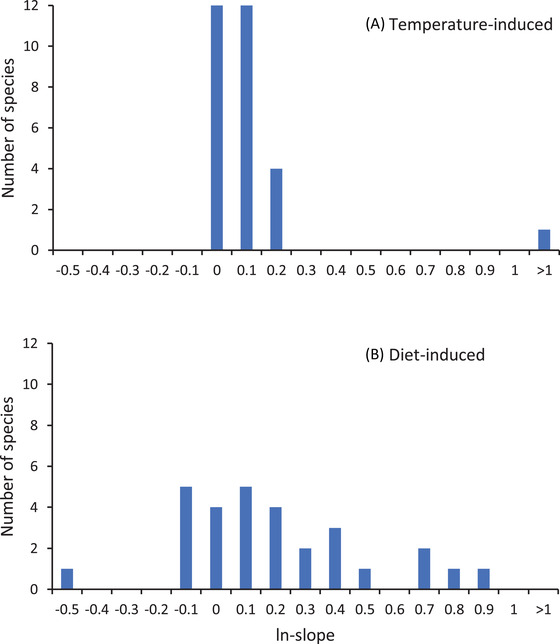
The distribution of individual effect sizes (logarithmically transformed RMA regression slopes of male development time on female development time = ln‐slopes) describing sex differences in (A) temperature‐ and (B) diet‐induced plasticity in development time. Note that ln‐slopes describing sex‐specific responses to temperature are mostly close to zero, referring to just a small sex difference in development time plasticity. By contrast, ln‐slopes quantifying sex differences in diet‐induced plasticity are much more widely scattered, indicating that diet‐induced reaction norms often substantially differ between the sexes. Data on sex differences in diet‐induced plasticity were obtained from the study of Teder et al. (2021). The single species displaying a strongly deviating pattern of sex‐specific thermal plasticity (panel A) was considered an outlier and disregarded in statistical analyses (see *Results*).

A pairwise comparison of sex‐specific reaction norms to temperature and diet indicated that diet typically induces more pronounced sex differences in plastic responses than temperature (permutation test‐based meta‐analysis: *P* < 0.001; the *P*‐value being obtained disregarding an outlier: see Fig. 2). Of the 30 species for which pairwise data were available, only in four species (13.3%) sex differences in development time responses were greater in experiments manipulating temperature (Fig. 2; Table S3). In addition to the pairwise analysis, we visually evaluated the frequency distributions of individual ln‐slopes describing sex differences in thermal plasticity with those quantifying sex differences in diet‐induced responses. To do so, the total sample of 199 primary datasets used in this study was compared to an analogous dataset from our earlier study (189 datasets, Teder et al. 2021). In consistence with the pairwise analysis, thermal ln‐slopes appeared to be strongly clustered around zero (Fig. S1A), whereas ln‐slopes describing responses to diet were widely scattered (Fig. S1B).

## Discussion

Unless tolerance limits are reached, insects develop faster under higher temperatures (Ratte 1984; Atkinson 1994; this study). The results of the present study suggest that such thermal response of development time tends to be broadly similar in males and females. Such an overall conclusion follows from two complementary observations. First, the proportions of datasets in which either males or females displayed higher plasticity were rather similar. Second, in a high fraction of cases, the change of female and male development times with temperature showed only a slight deviation from proportionality, especially when compared to sex differences in diet‐induced plastic responses. Our study, coupled with the consistent findings of an analogous synthesis on adult body sizes (Hirst et al. 2015), indicates that thermal sensitivity of both age and size at maturity does not differ notably for the two sexes.

Clearly, the argument of the similarity across the sexes does not imply that the thermal responses are strictly identical for males and females. Indeed, some of the (still scarce) case studies on particular species have found (mostly small but) significant differences (Fischer and Fiedler 2002; Stillwell and Fox 2007; Fischer et al. 2011). Instead, the conclusion about the relative invariability of the reaction norms should be understood against an appropriate background. Such a background is provided by the comparable reaction norms for size and time at maturity in response to food quality and quantity (Teder et al. 2014). Sex differences in these reaction norms have been found to be remarkably larger than in the thermal reaction norms considered in the present study (Teder and Tammaru 2005; Stillwell et al. 2010; Teder et al. 2021; this study).

Under the assumption of ecologically based optimality, we have good reasons to expect thermal responses to differ between males and females. This is because, in insects, both body size and development time are under intense ecological selective pressures, with these pressures being typically sex‐specific to a considerable extent. In many insects, female body size is under strong directional selection due to the fecundity advantage of large females (Honek 1993; Tammaru et al. 2002). Such a positive fitness effect is sometimes paralleled by the mate acquisition advantage of large males (Thornhill and Alcock 1983). However, overall, size dependence of fitness is typically weaker in males than in females (Charnov 1982), as reflected in the predominantly female‐biased sexual size dimorphism in insects (Teder and Tammaru 2005). On these premises, we should expect females to “care” more about attaining large size, and thus extend their development relatively more than males in conditions threatening with suboptimally small adult sizes. Other, case‐specific scenarios are definitely possible, but equal thermal sensitivity of males and females across species is hardly an expected outcome in the framework of optimality models, just because fitness functions of time and size are markedly different for the two sexes.

From a slightly different perspective, earlier works have shown that the sex with the larger trait values tends to be more plastic with respect to the trait in question (Teder and Tammaru 2005; Rohner et al. 2018). Accordingly, we expected sex differences in thermal sensitivity to be more pronounced in species with the larger sex difference in development time. This prediction was not met, however, and in fact, there was some evidence for an opposite trend, that is, sex differences in development time plasticity tended to decrease with increasing sex differences in development time.

The limited differences between the two sexes suggest that the evolvability of thermal responses in the reaction norms may be low, the shape of the reaction norms being primarily determined by slowly evolving physiological constraints rather than by responses to ecologically based selective pressures. This message is reinforced by the consistency of the patterns across insect orders with markedly different evolutionary history and ecology (Table 1). Nevertheless, alternative interpretations for our findings cannot be excluded a priori. For example, in principle, we cannot rule out ecologically based selective pressures that would favor equal relative response of developmental periods in the two sexes. If this was the case, the patterns documented in this study could indeed have an adaptive background. However, we cannot see in which ecological settings such selective pressures might appear, and especially how could they be general enough to apply to ecologically very different insect groups. We therefore stick to the interpretation that some physiologically based constraint on ecologically driven adaptive evolution of thermal reaction norms is the most plausible explanation of the limited sex differences in thermal plasticity.

At first glance, a constraint‐based explanation fits well into the broader context. The evolvability of traits characterizing the responses of organisms to their thermal environment (= thermal sensitivity) has traditionally been considered low (Hertz et al. 1983; Crowley 1985; van Damme et al. 1990; Hoffmann et al. 2013; Bennett et al. 2021). Nevertheless, broad generalizations may still be premature as different aspects of thermal sensitivity may display markedly different evolvabilities (Angilletta et al. 2002; Bodensteiner et al. 2020). Indeed, there is some evidence for high evolvability of such traits in microevolutionary contexts (e.g., Moghadam et al. 2020), and for insects, cases of rapid evolution of thermal reaction norms in the wild have been revealed (Kingsolver et al. 2007). By contrast, some comparable insect studies have found low heritabilities of thermal sensitivity (Klepsatel et al. 2013; Logan et al. 2020).

The picture appears to be even more fragmentary on the macroevolutionary scale, where the cross‐species studies on thermal reaction norms of developmental traits are relatively scarce, and tend to focus on aspects other than the temperature dependence of growth rates or developmental periods. For insects in particular, the few available comparative studies hint at evolutionary conservatism of developmental schedules: related species tend to have similar thermal reaction norms (Jarosik et al. 2011; Kutcherov 2016). Moreover, there is considerable indirect evidence of high conservatism of temperature‐related adaptations in insects. In particular, numerous insect clades are widespread in tropical regions but have failed to invade temperate areas in the course of tens of millions of years they have been around (e.g., Chazot et al. 2021), being presumably limited by climatic conditions at higher latitudes (Marshall et al. 2020). Naturally, the thermal environment is not the only factor that can preclude the northward spread. Nevertheless, systematic shifts in distribution limits of many species toward higher latitudes with the ongoing climate warming (Parmesan et al. 1999; Parmesan 2006; Breed et al. 2013; Tiitsaar et al. 2019) provide evidence of a major role of temperature in determining distribution ranges. The evolution of thermal tolerance in insects must be slow as it has not facilitated comparable shifts over thousands of years passed since the end of the ice age.

As different traits reflecting thermal plasticity may well have different evolvabilities (Angilletta et al. 2002), an evolutionary understanding of the responses of developmental schedules to temperature can benefit from decomposing the reaction norm to its elements (cf. van der Have and de Jong 1996; Forster and Hirst 2012). In particular, the duration of immature development can be seen as resulting from the values of two different traits: the (instantaneous) growth rate (Dmitriew 2011; Meister et al. 2018) and the timing of maturation (at which size growth will stop; Stearns 1992; Day and Rowe 2002; Davidowitz and Nijhout 2004; Teder et al. 2014). A constraint on instantaneous growth rate is intuitively easy to understand (based on the universal temperature dependence of enzyme kinetics: van der Have and de Jong 1996; Hochachka and Somero 2002; Arcus et al. 2016), especially because many insects maximize their growth rates within physiologically attainable limits (Kause et al. 1999; Tammaru et al. 2004). Our results suggest that there should also be constraints on the thermal plasticity of the maturation decision. Such a view is supported by careful studies on a model insect showing that the physiological mechanisms behind maturation decision have limited thermal plasticity, and do not differ between the two sexes (Davidowitz et al. 2004; Stillwell and Davidowitz 2010). As a particular implication, the emerging mechanistic view on the thermal reaction norm of body size determination in insects may be seen as supporting the nonadaptive explanations of the temperature‐size rule (Angilletta et al. 2004; Ghosh et al. 2013; Forster et al. 2011; Verberk et al. 2021). Indeed, in the light of the limited evolvability of thermal reaction norms, it is unlikely that the typically observed smaller body size of ectotherms in warmer conditions (Atkinson 1994) can be a response to ecologically based selective pressures.

Our results add a new dimension to the conclusion that sex differences in diet‐induced reaction norms are much more pronounced than those observed in response to different temperatures (Hirst et al. 2015, compared to Teder and Tammaru 2005). To explain this pattern, we may not need an explanation more complex than the overall principle of low evolvability of thermal sensitivity, discussed above. Some more specific considerations may nevertheless deserve attention. First, the limited food resources may not necessarily be equally limiting for the two sexes just due to the sex‐specific chemical composition of the adult body (Molleman et al. 2011; Back and King 2013; Kulma et al. 2019). This would imply that food manipulations that are technically equal for the two sexes may, in fact, imply different levels of nutritional stress for males and females. Second, in nature, it is perhaps a common scenario that an insect is exposed to certain nutritional conditions for its entire juvenile period (e.g., a lepidopteran larva feeding on a particular plant individual), whereas extreme thermal conditions have mostly a much more transient character. Adaptive plasticity in development is more likely to evolve in response to predictably long‐lasting environmental effects, and hence is more likely in response to food stress than thermal stress. It cannot thus be excluded that the explanation for the differences in responses to the gradients of diet and temperature also includes some adaptationist elements. Similarly, one cannot exclude that the slight tendency for an increase in the plasticity of male development time in species with strongly female‐biased sexual bimaturism may have an adaptive basis. Indeed, it should be adaptive for males to emerge not too far apart in time relative to females (for the evolution of protandry, see Wiklund and Fagerström 1977; Teder et al. 2021).

In conclusion, we found that the juveniles of male and female insects respond similarly to ambient temperatures. We propose that the most likely interpretation should invoke some constraints on thermal plasticity. This observation would provide guidance for further phylogenetic‐comparative studies on the subject. If confirmed, the low evolvability of thermal reaction norms of insect development should be considered in various applied contexts, particularly in predicting the evolutionary responses to climate warming (Chown et al. 2010; Hoffmann and Sgro 2011; Catullo et al. 2019; Weaving et al. 2022).

## CONFLICT OF INTEREST

The authors declare no conflict of interest.

## AUTHOR CONTRIBUTIONS

TTe conceived the ideas and designed methodology, participated in data collection and data analysis, and led the writing of the manuscript. KT participated in data collection, performed preliminary analyses, and wrote a part of the first draft. AK conducted the meta‐analysis and wrote a part of the first draft. TTa wrote a part of the first draft and made extensive contributions to the development, drafting, and editing of the manuscript. All authors gave final approval for publication.

## DATA ARCHIVING

Data and R scripts are available in the Dryad Repository (https://doi.org/10.5061/dryad.djh9w0w3q). New variables derived from literature data are an integral part of this study and are provided in the Supporting Information.

DATA SOURCES

Abdelrahman, I.

1974. Growth, development and innate capacity for increase in *Aphytis chrysomphali* Mercet and *A. melinus* DeBach, parasites of California red scale, *Aonidiella aurantii* (Mask.), in relation to temperature. Aust. J. Zool.
22:213–230.

Ahmadi, K.
, 
C.
Sengonca
, and 
P.
Blaeser

2007. Effect of two different temperatures on the biology of predatory flower bug *Orius similis* Zheng (Heteroptera: Anthocoridae) with two different aphid species as prey. Turk. J. Entomol.
31:253–268.

Ahn, J. J.
, 
K. S.
Choi
, and 
S.
Koh

2019. Effects of temperature on the development, fecundity, and life table parameters of *Riptortus pedestris* (Hemiptera: Alydidae). Appl. Entomol. Zool.
54:63–74.

Alcalay, Y.
, 
D.
Puzhevsky
, 
I.
Tsurim
, 
I.
Scharf
, and 
O.
Ovadia

2018. Interactive and sex‐specific life‐history responses of *Culex pipiens* mosquito larvae to multiple environmental factors. J. Zool.
306:268–278.

Allsopp, P. G.

1977. Biology and capacity for increase of *Monistria discrepans* (Walker) (Orthoptera: Pyrgomorphidae) in the laboratory. J. Aust. Ent. Soc.
16:207–213.

Allsopp, P. G.
, 
B. A.
Cowie
, and 
B. A.
Franzmann

1983. Development of immature stages of the lucerne leafroller *Merophyas divulsana* (Walker) (Lepidoptera: Tortricidae) under constant temperatures and on several larval diets. J. Aust. Ent. Soc.
22:287–291.

Amano, K.

1983. Studies on the intraspecific competition in dung‐breeding flies I. Effects of larval density on yellow dung fly, *Scatophaga stercoraria* L. (Diptera: Scatophagidae). Jpn J. Sanit. Zool.
34:165–175.

Amin, O. M.
, 
L.
Jun
, 
L.
Shangjun
, 
Z.
Yumei
, and 
S.
Liannzhi

1993. Development and longevity of *Nosopsyllus laeviceps kuzenkovi* (Siphonaptera) from Inner Mongolia under laboratory conditions. J. Parasitol.
79:193–197.8459329

Amoudi, M. A.

1993. Effect of temperature on the developmental stages of *Wohlfahrtia nuba* (Diptera: Sarcophagidae). J. Egypt. Soc. Parasitol.
23:697–705.8308345

Arakawa, R.
, and 
Y.
Namura

2002. Effects of temperature on development of three *Trissolcus* spp. (Hymenoptera: Scelionidae), egg parasitoids of the brown marmorated stink bug, *Halyomorpha halys* (Hemiptera: Pentatomidae). Entomol. Sci.
5:215–218.

Armbruster, P.
, and 
J. E.
Conn

2006. Geographic variation of larval growth in North American *Aedes albopictus* (Diptera: Culicidae). Ann. Entomol. Soc. Am.
99:1234–1243.

Atwal, A. S.

1955. Influence of temperature, photoperiod, and food on the speed of development, longevity, fecundity, and other qualities of the diamond‐back moth *Plutella maculipennis* (Curtis) (Lepidoptera: Tineidae). Aust. J. Zool.
3:185–221.

Barbosa, P.
, 
M.
Waldvogel
, 
P.
Martinat
, and 
L. W.
Douglass

1983. Developmental and reproductive performance of the gypsy moth, *Lymantria dispar* (L.) (Lepidoptera: Lymantriidae), on selected hosts common to mid‐Atlantic and southern forests. Environ. Entomol.
12:1858–1862.

Barbosa, P.
, 
P.
Martinat
, and 
M.
Waldvogel

1986. Development, fecundity and survival of the herbivore *Lymantria dispar* and the number of plant species in its diet. Ecol. Entomol.
11:1–6.

Bari, M. N.
, 
M.
Jahan
, and 
K. S.
Islam

2015. Effects of temperature on the life table parameters of *Trichogramma zahiri* (Hymenoptera: Trichogrammatidae), an egg parasitoid of *Dicladispa armigera* (Chrysomelidae: Coleoptera). Environ. Entomol.
44:368–378.2631319110.1093/ee/nvu028

Barker, J. E.
, 
G. M.
Poppy
, and 
C. C.
Payne

2006. Suitability of Arabidopsis thaliana as a model for host plant–*Plutella xylostella*–*Cotesia plutellae* interactions. Entomol. Exp. Appl.
122:17–26.

Barzegar, S.
, 
A. A.
Zamani
, 
S.
Abbasi
, 
R. V.
Shooshtari
, and 
N. S.
Farsani

2016. Temperature‐dependent development modeling of the phorid fly *Megaselia halterata* (Wood) (Diptera: Phoridae). Neotrop. Entomol.
45:507–517.2714722810.1007/s13744-016-0400-3

Bauerfeind, S. S.
, and 
K.
Fischer

2005. Effects of food stress and density in different life stages on reproduction in a butterfly. Oikos.
111:514–524.

Bauerfeind, S. S.
, and 
K.
Fischer

2009. Effects of larval starvation and adult diet‐derived amino acids on reproduction in a fruit‐feeding butterfly. Entomol. Exp. Appl.
130:229–237.

Bavaresco, A.
, 
M. S.
Garcia
, 
A. D.
Grützmacher
, and 
J. F. R.
Ringenberg

2002. Biology and thermal requirements of *Spodoptera cosmioides* (Walk.) (Lepidoptera: Noctuidae). Neotrop. Entomol.
31:49–54.

Bazzocchi, G. G.
, 
A.
Lanzoni
, 
G.
Burgio
, and 
M. R.
Fiacconi

2003. Effects of temperature and host on the pre‐imaginal development of the parasitoid *Diglyphus isaea* (Hymenoptera: Eulophidae). Biological Control.
26:74–82.

Bell, H. A.
, 
G. C.
Marris
, 
F.
Smethurst
, and 
J. P.
Edwards

2003. The effect of host stage and temperature on selected developmental parameters of the solitary endoparasitoid *Meteorus gyrator* (Thun.) (Hym., Braconidae). J. Appl. Entomol.
127:332–339.

Berner, D.
, 
W. U.
Blanckenhorn
, and 
C.
Körner

2005. Grasshoppers cope with low host plant quality by compensatory feeding and food selection: N limitation challenged. Oikos.
111:525–533.

Bezerra, C. E. S.
, 
P. K. A.
Tavares
, 
C. H. F.
Nogueira
, 
L. P. M.
Macedo
, and 
E. L.
Araujo

2012. Biology and thermal requirements of *Chrysoperla genanigra* (Neuroptera: Chrysopidae) reared on *Sitotroga cerealella* (Lepidoptera: Gelechiidae) eggs. Biol. Control.
60:113–118.

Blanckenhorn, W. U.
, and 
C.
Henseler

2005. Temperature‐dependent ovariole and testis maturation in the yellow dung fly. Entomol. Exp. Appl.
116:159–165.

Blanckenhorn, W. U.

1997. Altitudinal life history variation in the dung flies *Scathophaga stercoraria* and *Sepsis cynipsea*
. Oecologia.
109:342–352.2830753010.1007/s004420050092

Blanckenhorn, W. U.

1997. Effects of temperature on growth, development and diapause in the yellow dung fly ‐ against all the rules?
Oecologia.
111:318–324.2830812510.1007/s004420050241

Blanckenhorn, W. U.
, and 
A.
Heyland

2004. The quantitative genetics of two life history trade‐offs in the yellow dung fly in abundant and limited food environments. Evol. Ecol.
18:385–402.

Bommireddy, P. L.
, 
M. N.
Parajulee
, and 
D. O.
Porter

2004. Influence of constant temperatures on life history of immature *Lygus elisus* (Hemiptera: Miridae). Environ. Entomol.
33:1549–1553.

Bonte, J.
, 
D.
Vangansbeke
, 
S.
Maes
, 
M.
Bonte
, 
D.
Conlong
, and 
P.
De Clercq

2012. Moisture source and diet affect development and reproduction of *Orius thripoborus* and *Orius naivashae*, two predatory anthocorids from southern Africa. J. Insect Sci.
12:1.10.1673/031.012.0101PMC346593222935002

Bonte, J.
, 
M.
De Ro
, 
D.
Conlong
, and 
P.
De Clercq

2012. Thermal biology of the predatory bugs *Orius thripoborus* and *O. naivashae* (Hemiptera: Anthocoridae). Environ. Entomol.
41:989–996.

Brakefield, P. M.
, and 
V.
Mazzotta

1995. Matching field and laboratory environments: effects of neglecting daily temperature variation on insect reaction norms. J. Evol. Biol.
8:559–573.

Buei, K.
, 
S. H.
Park
, and 
H.
Yamugi

1978. Bionomics of three species of fleshflies, *Boettcherisca peregrina*, *Parasarcophaga similis* and *P. crassipalpis*, with reference to the effects of temperature on the development and fecundity. Jpn. J. Sanit. Zool.
29:125–132.

Burke, S.
, 
A. S.
Pullin
, 
R. J.
Wilson
, and 
C. D.
Thomas

2005. Selection for discontinuous life‐history traits along a continuous thermal gradient in the butterfly Aricia agestis. Ecol. Entomol.
30:613–619.

Cave, R. D.
, 
C.
Sciacchetano
, and 
R.
Diaz

2009. Temperature‐dependent development of the cycad aulacaspis scale, *Aulacaspis yasumatsui* (Hemiptera: Diaspididae). Fla. Entomol.
92:578–581.

Charles, J. G.
, 
J. M.
Kean
, and 
A.
Chhagan

2006. Developmental parameters and voltinism of the painted apple moth, *Teia anartoides* Walker (Lepidoptera: Lymantriidae) in New Zealand. N. Z. Entomol.
29:27–36.

Chen, C.
, 
Q. ‐ W.
Xia
, 
H. ‐ J.
Xiao
, 
L.
Xiao
, and 
F. ‐ S.
Xue

2014. A comparison of the life‐history traits between diapause and direct development individuals in the cotton bollworm, *Helicoverpa armigera*
. J. Insect Sci.
14:19.2537316610.1093/jis/14.1.19PMC4199537

Chong, J. ‐ H.
, 
R. D.
Oetting
, and 
M. W.
van Iersel

2003. Temperature effects on the development, survival, and reproduction of the Madeira mealybug, *Phenacoccus madeirensis* Green (Hemiptera: Pseudococcidae), on Chrysanthemum. Ann. Entomol. Soc. Am.
96:539–543.

Chong, J. ‐ H.
, 
A. L.
Roda
, and 
C. M.
Mannion

2008. Life history of the mealybug, *Maconellicoccus hirsutus* (Hemiptera: Pseudococcidae), at constant temperatures. Environ. Entomol.
37:323–332.1841990310.1603/0046-225X(2008)37[323:LHOTMM]2.0.CO;2

Combs, R. L.
, and 
J. R.
Valerio

1980. Biology of the fall armyworm on four varieties of bermudagrass when held at constant temperatures. Environ. Entomol.
9:393–396.

Silva, C. A. D.

2004. Efeitos da temperatura no desenvolvimento, fecundidade e longevidade de *Gargaphia torresi* Lima (Hemiptera, Tingidae). Rev. Bras. Entomol.
48:547–552.

De Block, M.
, and 
R.
Stoks

2003. Adaptive sex‐specific life history plasticity to temperature and photoperiod in a damselfly. J. Evol. Biol.
16:986–995.1463591410.1046/j.1420-9101.2003.00581.x

Del Pino, M.
, 
T.
Cabello
, and 
E.
Hernandez‐Suarez

2020. Age‐stage, two‐sex life table of *Chrysodeixis chalcites* (Lepidoptera: Noctuidae) at constant temperatures on semi‐synthetic diet. Environ. Entomol.
49:777–788.3240691110.1093/ee/nvaa050

Diamond, S. E.
, and 
J. G.
Kingsolver

2010. Environmental dependence of thermal reaction norms: host plant quality can reverse the temperature‐size rule. Am. Nat.
175:1–10.1991198410.1086/648602

Doganlar, O.

2008. Temperature‐dependent development and degree‐day model of European leaf roller, *Archips rosanus*
. J. Plant Prot. Res.
48:63–72.

Farjana, T.
, 
N.
Tuno
, and 
Y.
Higa

2012. Effects of temperature and diet on development and interspecies competition in *Aedes aegypti* and *Aedes albopictus*
. Med. Vet. Entomol.
26:210–217.2178113910.1111/j.1365-2915.2011.00971.x

Farsani, N. S.
, 
A. A.
Zamani
, 
S.
Abbasi
, and 
K.
Kheradmand

2013. Effect of temperature and button mushroom varieties on life history of *Lycoriella auripila* (Diptera: Sciaridae). J. Econ. Entomol.
106:115–123.2344802210.1603/ec12241

Ferreira de Almeida, M. A.
, 
A.
Pires do Prado
, and 
C. J.
Geden

2002. Influence of temperature on development time and longevity of *Tachinaephagus zealandicus* (Hymenoptera: Encyrtidae), and effects of nutrition and emergence order on longevity. Environ. Entomol.
31:375–380.

Fischer, K.
, and 
K.
Fiedler

2000. Sex‐related differences in reaction norms in the butterfly *Lycaena tityrus* (Lepidoptera: Lycaenidae). Oikos.
90:372–380.

Fischer, K.
, and 
K.
Fiedler

2000. Response of the copper butterfly *Lycaena tityrus* to increased leaf nitrogen in natural food plants: evidence against the nitrogen limitation hypothesis. Oecologia.
124:235–241.2830818410.1007/s004420000365

Fischer, K.
, and 
K.
Fiedler

2001. Dimorphic growth patterns and sex‐specific reaction norms in the butterfly *Lycaena hippothoe sumadiensis*
. J. Evol. Biol.
14:210–218.

Fischer, K.
, and 
K.
Fiedler

2002. Reaction norms for age and size at maturity in response to temperature: a test of the compound interest hypothesis. Evol. Ecol.
16:333–349.

Fischer, K.
, 
P. M.
Brakefield
, and 
B. J.
Zwaan

2003. Plasticity in butterfly egg size: why larger offspring at lower temperatures?
Ecology.
84:3138–3147.

Folgarait, P. J.
, 
M. G.
Chirino
, 
R. J.
Wilson Patrock
, and 
L. E.
Gilbert

2005. Development of *Pseudacteon obtusus* (Diptera: Phoridae) on *Solenopsis invicta* and *Solenopsis richteri* fire ants (Hymenoptera: Formicidae). Environ. Entomol.
34:308–316.10.1603/0022-0493-99.2.29516686126

Forsberg, J.
, and 
C.
Wiklund

1988. Protandry in the green‐veined white butterfly, *Pieris napi* L. (Lepidoptera; Pieridae). Funct. Ecol.
2:81–88.

Francis, A. W.
, 
M. T. K.
Kairo
, and 
A. L.
Roda

2012. Developmental and reproductive biology of *Planococcus minor* (Hemiptera: Pseudococcidae) under constant temperatures. Fla. Entomol.
95:297–303.

Frouz, J.
, 
A.
Ali
, and 
R. J.
Lobinske

2002. Influence of temperature on developmental rate, wing length, and larval head capsule size of pestiferous midge *Chironomus crassicaudatus* (Diptera: Chironomidae). J. Econ. Entomol.
95:699–705.1221680910.1603/0022-0493-95.4.699

Fukuda, T.
, 
S.
Wakamura
, 
N.
Arakaki
, and 
K.
Yamagishi

2007. Parasitism, development and adult longevity of the egg parasitoid *Telenomus nawai* (Hymenoptera: Scelionidae) on the eggs of *Spodoptera litura* (Lepidoptera: Noctuidae). Bull. Entomol. Res, 97:185–190.1741148110.1017/S0007485307004841

Gautam, S. G.
, 
G. P.
Opit
, and 
K. L.
Giles

2010. Population growth and development of the psocid *Liposcelis rufa* (Psocoptera: Liposcelididae) at constant temperatures and relative humidities. J. Econ. Entomol.
103:1920–1928.2106199710.1603/ec10127

Gautam, S. G.
, 
G. P.
Opit
, and 
K.
Shakya

2016. Population growth and development of the psocid *Liposcelis fusciceps* (Psocoptera: Liposcelididae) at constant temperatures and relative humidities. Environ. Entomol.
45:237–244.2638593110.1093/ee/nvv148

Geden, C. J.
, 
M. A.
Ferreira de Almeida
, and 
A.
Pires do Prado

2003. Effects of Nosema disease on fitness of the parasitoid *Tachinaephagus zealandicus* (Hymenoptera: Encyrtidae). Environ. Entomol.
32:1139–1145.

Gillespie, D. R.
, and 
R. R.
McGregor

2000. The functions of plant feeding in the omnivorous predator *Dicyphus hesperus*: water places limits on predation. Ecol. Entomol.
25:380–386.

Gillespie, D. R.
, 
J. A. S.
Sanchez
, and 
R. R.
McGregor

2004. Cumulative temperature requirements and development thresholds in two populations of *Dicyphus hesperus* (Hemiptera: Miridae). Can. Entomol.
136:675–683.

Golizadeh, A.
, and 
M. P.
Zalucki

2012. Estimating temperature‐dependent developmental rates of potato tuberworm, *Phthorimaea operculella* (Lepidoptera: Gelechiidae). Insect Science.
19:609–620.

Gomi, T.

2006. Sexual difference in the effect of temperature on the larval development in *Hyphantria cunea* (Drury) (Lepidoptera: Arctiidae). Appl. Entomol. Zool.
41:303–307.

Gotoh, T.
, 
M.
Koyama
, 
Y.
Hagino
, and 
K.
Doke

2011. Effect of leaf toughness and temperature on development in the lilac pyralid, *Palpita nigropunctalis* (Bremer) (Lepidoptera: Crambidae). J. Asia Pac. Entomol.
14:173–178.

Graeve, I.

2008. Degree days and phenological synchrony in Western tussock moth and coast live oak. Master's thesis, San Jose State University, San Jose, CA.

Greenberg, S. M.
, 
M.
Setamou
, 
T. W.
Sappington
, 
T. ‐ X.
Liu
, 
R. J.
Coleman
, and 
J. S.
Armostrong

2005. Temperature‐dependent development and reproduction of the boll weevil (Coleoptera: Curculionidae). Insect Science.
12:449–459.

Guzman, L. I.
, and 
A. M.
Frake

2007. Temperature affects *Aethina tumida* (Coleoptera: Nitidulidae) development. J. Apic. Res.
46:88–93.

Hamilton, J. G.
, and 
M. P.
Zalucki

1991. Effect of temperature on development rate, survival and fecundity of cotton tipworm, *Croidosema plebejana* Zeller (Lepidoptera: Tortricidae). Aust. J. Zool.
39:191–200.

Hamilton, J. G.
, and 
M. P.
Zalucki

1993. Interactions between a specialist herbivore, *Crocidosema plebejana*, and its host plants *Malva parviflora* and cotton, *Gossypium hirsutum*: larval performance. Entomol. Exp. Appl.
66:199–205.

Harries, F. H.
, and 
J. R.
Douglass

1948. Bionomic studies on the beet leafhopper. Ecol. Monogr.
18:45–79.

He, X.
, 
Q.
Wang
, and 
A.
Carpenter

2003. Thermal requirements for the development and reproduction of *Nysius huttoni* White (Heteroptera: Lygaeidae). J. Econ. Entomol.
96:1119–1125.1450358210.1093/jee/96.4.1119

Hill, J. K.
, and 
A. G.
Gatehouse

1992. Effects of temperature and photoperiod on development and pre‐reproductive period of the silver Y moth *Autographa gamma* (Lepidoptera: Noctuidae). Bull. Entomol. Res.
82:335–341.

Hoang, L. K.
, and 
K.
Takasu

2005. Helicoverpa armigera as an alternative host of the larval parasitoid *Microplitis croceipes* (Hymenoptera: Braconidae). Appl. Entomol. Zool.
40:679–686.

Hoddle, M. S.

2002. Developmental and reproductive biology of *Scirtothrips perseae* (Thysanoptera: Thripidae): a new avocado pest in California. Bull. Entomol. Res.
92:279–285.1219143510.1079/BER2002169

Horgan, F. G.
, 
D. T.
Quiring
, 
A.
Lagnaoui
, and 
Y.
Pelletier

2012. Life histories and fitness of two tuber moth species feeding on native Andean potatoes. Neotrop. Entomol.
41:333–340.2395007010.1007/s13744-012-0042-z

Horgan, F. G.
, 
D. T.
Quiring
, 
A.
Lagnaoui
, 
A. R.
Salas
, and 
Y.
Pelletier

2007. Periderm‐ and cortex‐based resistance to tuber‐feeding *Phthorimaea operculella* in two wild potato species. Entomol. Exp. Appl.
125:249–258.

Hou, Y.
, and 
Z.
Weng

2010. Temperature‐dependent development and life table parameters of *Octodonta nipae* (Coleoptera: Chrysomelidae). Environ. Entomol.
39:1676–1684.2254646710.1603/EN10015

Huang, X. ‐ L.
, 
L.
Xiao
, 
H. ‐ M.
he
, and 
F. ‐ S.
Xue

2018. Effect of rearing conditions on the correlation between larval development time and pupal weight of the rice stem borer, *Chilo suppressalis*
. Ecol. E.
8:12694–12701.10.1002/ece3.4697PMC630889830619574

Ichiki, R.
, and 
S.
Nakamura

2007. Oviposition and immature development of the parasitoid fly *Compsilura concinnata* (Meigen) (Diptera: Tachinidae). Jpn. Agric. Res. Q.
41:227–232.

Ichiki, R.
, 
K.
Takasu
, and 
H.
Shima

2003. Effects of temperature on immature development of the parasitic fly *Bessa parallela* (Meigen) (Diptera: Tachinidae). App. Entomol. Zool.
38:435–439.

Iltis, C.
, 
P.
Louapre
, 
K.
Pecharova
, 
D.
Thiery
, 
S.
Zito
, 
B.
Bois
, et al. 2019. Are life‐history traits equally affected by global warming? A case study combining a multi‐trait approach with fine‐grain climate modeling. J. Insect Physiol.
117:103916.3134439110.1016/j.jinsphys.2019.103916

Ishijima, C.
, 
Y.
Sato
, and 
M.
Ohtaishi

2008. Effect of temperature and host on the development, sex ratio, emergence rate and body size of *Trichogramma dendrolimi* Matsumura (Hymenoptera: Trichogrammatidae), an egg parasitoid of the tea tortrix. Appl. Entomol. Zool.
52:193–200.

Jang, T.
, 
M. S.
Rho
, 
S. ‐ H.
Koh
, and 
K. P.
Lee

2015. Host–plant quality alters herbivore responses to temperature: a case study using the generalist *Hyphantria cunea*
. Entomol. Exp. Appl.
154:120–130.

Johnson, T.
, and 
J. H.
Giliomee

2011. Development of the oleander mealybug, *Paracoccus burnerae* (Brain) (Hemiptera: Pseudococcidae), on citrus at five temperatures. Afr. Entomol.
19:641–649.

Jones, J. M.
, and 
F. M.
Stephen

1994. Effect of temperature on development of hymenopterous parasitoids of *Dendroctonus frontalis* (Coleoptera: Scolytidae). Environ. Entomol.
23:457–463.

Joseph, G.
, and 
R. G.
Kelsey

1994. Acceptability and suitability of douglas‐fir as a secondary host for gypsy moth (Lepidoptera: Lymantriidae). Environ. Entomol.
23:396–405.

Kalaitzaki, A. P.
, 
D. P.
Lykouressis
, 
D. C.
Perdikis
, and 
V. Z.
Alexandrakis

2007. Effect of temperature on development and survival of the parasitoid *Pnigalio pectinicornis* (Hymenoptera: Eulophidae) reared on *Phyllocnistis citrella* (Lepidoptera: Gracillariidae). Environ. Entomol.
36:497–505.1754005610.1603/0046-225x(2007)36[497:eotoda]2.0.co;2

Karamaouna, F.
, and 
M. J. W.
Copland

2000. Host suitability, quality and host size preference of *Leptomastix epona* and *Pseudaphycus flavidulus*, two endoparasitoids of the mealybug *Pseudococcus viburni*, and host size effect on parasitoid sex ratio and clutch size. Entomol. Exp. Appl.
96:149–158.

Karamaouna, F.
, and 
M. J.
Copland

2009. Fitness and life history parameters of *Leptomastix epona* and *Pseudaphycus flavidulus*, two parasitoids of the obscure mealybug *Pseudococcus viburni*
. BioControl.
54:65–76.

Karolewski, P.
, 
J.
Grzebyta
, 
J.
Oleksyn
, and 
M. J.
Gietrych

2007. Temperature affects performance of *Lymantria dispar* larvae feeding on leaves of *Quercus robur*
. Dendrobiology.
58:43–49.

Kemmochi, T.
, 
S.
Fujimori
, and 
T.
Saito

2016. The leafminer *Liriomyza trifolii* (Diptera: Agromyzidae) encapsulates its koinobiont parasitoid *Halticoptera circulus* (Hymenoptera: Pteromalidae): implications for biological control. Bull. Entomol. Res.
106:322–327.2663984110.1017/S0007485315000930

Khan, M.
, 
P.
Gregg
, and 
R.
Mensah

2009. Effect of temperature on the biology of *Creontiades dilutus* (Stål) (Heteroptera: Miridae). Aust. J. Entomol.
48:210–216.

Kingsolver, J. G.
, 
G. J.
Ragland
, and 
S. E.
Diamond

2009. Evolution in a constant environment: thermal fluctuations and thermal sensitivity of laboratory and field populations of *Manduca sexta*
. Evolution; Internation Journal of Organic Evolution.
63:537–541.10.1111/j.1558-5646.2008.00568.x19154355

Kitajima, H.
, 
H.
Sakata
, 
S.
Kunitomo
, and 
Y.
Kawashima

2016. Effects of temperature on the development of *Diomea cremata* (Lepidoptera, Noctuidae). Jpn. J. Appl. Entomol. Zool.
60:205–209.

Kivan, M.
, and 
N.
Kilic

2006. Age‐specific fecundity and life table of *Trissolcus semistriatus*, an egg parasitoid of the sunn pest *Eurygaster integriceps*
. Entomol. Sci.
9:39–46.

Kivelä, S. M.
, 
P.
Välimäki
, and 
M. I.
Mäenpää

2012. Genetic and phenotypic variation in juvenile development in relation to temperature and developmental pathway in a geometrid moth. J. Evol. Biol.
25:881–891.2235664910.1111/j.1420-9101.2012.02478.x

Krasnov, B. R.
, 
I. S.
Khoklova
, 
L. J.
Fielden
, and 
N. V.
Burdelova

2001. Development rates of two *Xenopsylla* flea species in relation to air temperature and humidity. Med. Veter. Entomol.
15:249–258.10.1046/j.0269-283x.2001.00295.x11583441

Kruse, J. J.
, and 
K. F.
Raffa

1997. Effects of selected midwestern larval host plants on performance by two strains of the gypsy moth (Lepidoptera: Lymantriidae) parasitoid *Cotesia melanoscela* (Hymenoptera: Braconidae). Environ. Entomol.
26:1155–1166.

Lance, D. R.
, 
J. S.
Elkinton
, and 
C. P.
Schwalbe

1986. Feeding rhythms of gypsy moth larvae: effect of food quality during outbreaks. Ecology.
67:1650–1654.

Larios, G. L. B.
, 
K.
Ohno
, and 
F.
Fukuhara

2007. Effects of photoperiod and temperature on preimaginal development and summer diapause of *Chrysocharis pubicornis* (Zetterstedt) (Hymenoptera: Eulophidae), a pupal parasitoid of leafminers (Diptera: Agromyzidae). Appl. Entomol. Zool.
42:189–197.

Lauziere, I.
, 
M.
Setamou
, 
J.
Legaspi
, and 
W.
Jones

2002. Effect of temperature on the life cycle of *Lydella jalisco* (Diptera: Tachinidae), a Parasitoid of *Eoreuma loftini* (Lepidoptera: Pyralidae). Environ. Entomol.
31:432–437.

Lazarević, J.
, 
V.
Perić‐Mataruga
, 
B.
Stojković
, and 
N.
Tucić

2002. Adaptation of the gypsy moth to an unsuitable host plant. Entomol. Exp. Appl.
102:75–86.

Legaspi, J. C.
, and 
B. C.
Legaspi

2005. Life table analysis for *Podisus maculiventris* immatures and female adults under four constant temperatures. Environ. Entomol.
34:990–998.

Legaspi, J. C.
, 
B. C.
Legaspi
, 
A. M.
Simmons
, and 
M.
Soumare

2008. Life table analysis for immatures and female adults of the predatory beetle, *Delphastus catalinae*, feeding on whiteflies under three constant temperatures. J. Insect Sci.
8
7.2034529510.1673/031.008.0701PMC3061575

Lehtovaara, V. J.
, 
H.
Roininen
, and 
A.
Valtonen

2018. Optimal temperature for rearing the edible *Ruspolia differens* (Orthoptera: Tettigoniidae). J. Econ. Entomol.
111:2652–2659.3012490010.1093/jee/toy234

Lindroth, R. L.
, 
K. A.
Klein
, 
J. D. C.
Hemming
, and 
A. M.
Feuker

1997. Variation in temperature and dietary nitrogen affect performance of the gypsy moth (*Lymantria dispar* L.). Physiol. Entomol.
22:55–64.

Liu, Y. H.
, and 
J. H.
Tsai

2002. Effect of temperature on development, survivorship, and fecundity of *Lysiphlebia mirzai* (Hymenoptera: Aphidiidae), a parasitoid of *Toxoptera citricida* (Homoptera: Aphididae). Environ. Entomol.
31:418–424.

Liu, J. ‐ F.
, 
M.
Liu
, 
M. ‐ F.
Yang
, 
D. C.
Kontodimas
, 
X. ‐ F.
Yu
, and 
Q. ‐ X.
Lian

2014. Temperature‐dependent development of *Lista haraldusalis* (Walker) (Lepidoptera: Pyralidae) on *Platycarya strobilacea*
. J. Asia Pac. Entomol.
17:803–810.

Lopatina, E. B.
, and 
I. A.
Gusev

2019. A novel form of phenotypic plasticity of the thermal reaction norms for development in the bug *Graphosoma lineatum* (L.) (Heteroptera, Pentatomidae). Entomol. Rev.
99:417–436.

Luo, S.
, 
F.
Zhang
, and 
K.
Wu

2015. Effect of temperature on the reproductive biology of *Peristenus spretus* (Hymenoptera: Braconidae), a biological control agent of the plant bug *Apolygus lucorum* (Hemiptera: Miridae). Biocontrol Sci. Technol.
25:1410–1425.

Lysyk, T. J.

2001. Relationships between temperature and life history parameters of *Muscidifurax raptorellus* (Hymenoptera: Pteromalidae). Environ. Entomol.
30:982–992.

Lyytinen, A.
, 
L.
Lindström
, and 
J.
Mappes

2008. Genetic variation in growth and development time under two selection regimes in *Leptinotarsa decemlineata*
. Entomol. Exp. Appl.
127:157–167.

Mafi, S.
, and 
N.
Ohbayashi

2010. Biology of *Chrysocharis pentheus*, an endoparasitoid wasp of the citrus leafminer *Phyllocnistis citrella* Stainton. J. Agric. Sci. Technol.
12:145–154.

Mahdian, K.
, 
J.
Kerckhove
, 
L.
Tirry
, and 
P.
De Clercq

2006. Effects of diet on development and reproduction of the predatory pentatomids *Picromerus bidens* and *Podisus maculiventris*
. BioControl.
51:725–739.

Mahmood, A. R.
, 
S. S.
Liu
, 
Z. H.
Shi
, 
X. H.
Song
, and 
M. P.
Zalucki

2003. Lack of intraspecific biological variation between two geographical populations of *Oomyzus sokolowskii* (Hymenoptera: Eulophidae), a gregarious larval–pupal parasitioid of *Plutella xylostella* (Lepidoptera: Plutellidae). Bull. Entomol. Res.
93:169–177.1515329910.1079/ber2003284

Malekmohammadi, A.
, 
P.
Shishehbor
, and 
F.
Kocheili

2012. Influence of constant temperatures on development, reproduction and life table parameters of *Encarsia inaron* (Hymenoptera: Aphelinidae) parasitizing *Neomaskellia andropogonis* (Hemiptera: Aleyrodidae). Crop Protection.
34:1–5.

Mao, H.
, and 
Y.
Kunimi

1990. Effects of temperature and photoperiod on development of the oriental tea tortrix *Homona magnanima* Diakonoff (Lepidoptera: Tortricidae). Jpn. J. Appl. Entomol. Zool.
34:127–130.

Mawela, K. V.
, 
R.
Kfir
, and 
K.
Krüger

2013. Effect of temperature and host species on parasitism, development time and sex ratio of the egg parasitoid *Trichogrammatoidea lutea* Girault (Hymenoptera: Trichogrammatidae). Biological Control.
64:211–216.

McDonald, R. S.
, and 
J. H.
Borden

1995. Protandry in *Delia antiqua* (Diptera: Anthomyiidae). Ann. Entomol. Soc. Am.
88:756–763.

Milonas, P. G.
, and 
M.
Savopoulou‐Soultani

2000. Development, survivorship, and reproduction of *Adoxophyes orana* (Lepidoptera: Tortricidae) at constant temperaturesAnn. Entomol. Soc. Am.
93:96–102.

Milosavljevic, I.
, 
K. A.
McCalla
, 
D. A.
Ratkowsky
, and 
M. S.
Hoddle

2019. Effects of constant and fluctuating temperatures on development rates and longevity of *Diaphorencyrtus aligarhensis* (Hymenoptera: Encyrtidae). J. Econ. Entomol.
112:1062–1072.3068991610.1093/jee/toy429

Mirhosseini, M. A.
, 
Y.
Fathipour
, 
M.
Soufbaf
, and 
G. V. P.
Reddy

2018. Thermal requirements and development response to constant temperatures by *Nesidiocoris tenuis* (Hemiptera: Miridae), and implications for biological control. Environ. Entomol.
47:467–476.2952209410.1093/ee/nvy020

Mo, J.
, 
M.
Glover
, 
S.
Munro
, and 
A. C.
Beattie

2006. Development of *Epiphyas postvittana* (Lepidoptera: Tortricidae) on leaves and fruit of orange trees. J. Econ. Entomol.
99:1321–1326.1693768810.1603/0022-0493-99.4.1321

Morales‐Ramos, J. A.
, and 
M. G.
Rojas

2017. Temperature‐dependent biological and demographic parameters of *Coleomegilla maculata* (Coleoptera: Coccinellidae). J. Insect Sci.
17:55.2842341910.1093/jisesa/iex028PMC5416751

Myers, H. M.
, 
J. K.
Tomberlin
, 
B. D.
Lambert
, and 
D.
Kattes

2008. Development of black soldier fly (Diptera: Stratiomyidae) larvae fed dairy manure. Environ. Entomol.
37:11–15.1834879110.1603/0046-225x(2008)37[11:dobsfd]2.0.co;2

Nabeta, F. H.
, 
M.
Nakai
, and 
Y.
Kunimi

2005. Effects of temperature and photoperiod on the development and reproduction of *Adoxophyes honmai* (Lepidoptera: Tortricidae). Appl. Entomol. Zool.
40:231–238.

Nieuwenhove, G. A.
, 
E. A.
Frias
, and 
E. G.
Virla

2016. Effects of temperature on the development, performance and fitness of the corn leafhopper *Dalbulus maidis* (DeLong) (Hemiptera: Cicadellidae): implications on its distribution under climate change. Agric. For. Entomol.
18:1–10.

Noor‐ul‐Ane, M.
, and 
C.
Jung

2020. Temperature‐dependent development and survival of small hive beetle, *Aethina tumida* (Coleoptera: Nitidulidae). J. Apic. Res.
59:807–816.

Ohta, I.

2001. Effect of temperature on development of *Orius strigicollis* (Heteroptera: Anthocoridae) fed on *Frankliniella occidentalis* (Thysanoptera: Thripidae). Appl. Entomol. Zool.
36:483–488.

Okada, K.
, and 
T.
Miyatake

2007. 
*Librodor japonicus* (Coleoptera: Nitidulidae) life history, effect of temperature on development, and seasonal abundance. Appl. Entomol. Zool.
42:411–417.

Ottenheim, M. M.
, 
A. D.
Volmer
, and 
G. J.
Holloway

1996. The genetics of phenotypic plasticity in adult abdominal colour pattern of *Eristalis arbustorum* (Diptera: Syrphidae). Heredity.
77:493–499.

Pakyari, H.
, 
Y.
Fathipour
, and 
A.
Enkegaard

2011. Estimating development and temperature thresholds of *Scolothrips longicornis* (Thysanoptera: Thripidae) on eggs of two‐spotted spider mite using linear and nonlinear models. J. Pest Sci.
84:153–163.

Pandey, S.
, and 
R.
Singh

1999. Host size induced variation in progeny sex ratio of an aphid parasitoid *Lysiphlebia mirzai*
. Entomol. Exp. Appl.
90:61–67.

Panizzi, A. R.

1992. Performance of *Piezodorus guildinii* on four species of Indigofera legumes. Entomol. Exp. Appl.
63:221–228.

Pappas, M. L.
, 
E.
Karagiorgou
, 
G.
Papaioannou
, 
D. S.
Koveos
, and 
G. D.
Broufas

2013. Developmental temperature responses of *Chrysoperla agilis* (Neuroptera: Chrysopidae), a member of the European carnea cryptic species group. Biological Control.
64:291–298.

Prasad, Y. G.
, 
M.
Prabhakar
, 
G.
Sreedevi
, 
G.
Ramachandra Rao
, and 
B.
Venkateswarlu

2012. Effect of temperature on development, survival and reproduction of the mealybug, *Phenacoccus solenopsis* Tinsley (Hemiptera: Pseudococcidae) on cotton. Crop Protection.
39:81–88.

Reed, D. A.
, 
F.
Ganjisaffar
, 
J. C.
Palumbo
, and 
T. M.
Perring

2017. Effects of temperatures on immature development and survival of the invasive stink bug *Bagrada hilaris* (Hemiptera: Pentatomidae). J. Econ. Entomol.
110:2497–2503.2912120610.1093/jee/tox289

Rizvi, S. Z. M.
, 
A.
Raman
, 
W. M.
Wheatley
, and 
G.
Cook

2016. Oviposition preference and larval performance of *Epiphyas postvittana* (Lepidoptera: Tortricidae) on *Botrytis cinerea* (Helotiales: Sclerotiniaceae) infected berries of *Vitis vinifera* (Vitales: Vitaceae). Insect Science.
23:313–325.2542072010.1111/1744-7917.12191

Rohde, K.
, 
E.
Dreier
, and 
A.
Hochkirch

2015. Sex‐specific phenotypic plasticity in response to the trade‐off between developmental time and body size supports the dimorphic niche hypothesis. Biol. J. Linn. Soc.
115:48–57.

Sakashita, T.
, 
F.
Nakasuji
, and 
K.
Fujisaki

1997. Effects of temperature and photoperiod on nymphal development of the stink bug, *Pyrrhocoris sibiricus* Kuschakewitsch (Heteroptera: Pyrrhocoridae). Appl. Entomol. Zool.
32:153–157.

Sarfraz, M.
, 
L. M.
Dosdall
, and 
B. A.
Keddie

2007. Resistance of some cultivated Brassicaceae to infestations by *Plutella xylostella* (Lepidoptera: Plutellidae). J. Econ. Entomol.
100:215–224.1737083110.1603/0022-0493(2007)100[215:roscbt]2.0.co;2

Sarfraz, M.
, 
L. M.
Dosdall
, and 
B. A.
Keddie

2010. Performance of the specialist herbivore *Plutella xylostella* (Lepidoptera: Plutellidae) on Brassicaceae and non‐Brassicaceae species. Can. Entomol.
142:24–35.

Schoeller, E. N.
, and 
R. A.
Redak

2018. Temperature‐dependent development and survival of giant whitefly *Aleurodicus dugesii* (Hemiptera: Aleyrodidae) under constant temperatures. Environ. Entomol.
47:1586–1595.3018898810.1093/ee/nvy130

Shimoji, Y.

2011. Effect of temperature on the development of the West Indian sweet potato weevil, *Euscepes postfasciatus* (Fairmaire) (Coleoptera: Curculionidae) on an artificial diet. Appl. Entomol. Zool.
46:1–54.

Singh, P.
, 
E.
van Bergen
, 
O.
Brattström
, 
D.
Osbaldeston
, 
P. M.
Brakefield
, and 
V.
Oostra

2020. Complex multi‑trait responses to multivariate environmental cues in a seasonal butterfly. Evol. Ecol.
34:713–734.

Sithole, R.
, 
B.
Löhr
, and 
P.
Tagwireyi

2017. The influence of temperature on life history traits of *Diadegma mollipla* (Hymenoptera: Ichneumonidae), an African parasitoid of the diamondback moth, *Plutella xylostella* (Lepidoptera: Plutellidae). BioControl.
62:603–612.

Skovgard, H.
, and 
G.
Nachman

2016. Temperature‐ and age‐dependent survival, development, and oviposition rates of the pupal parasitoid *Spalangia cameroni* (Hymenoptera: Pteromalidae). Environ. Entomol.
45:1063–1075.2729839210.1093/ee/nvw055

Smith, A. M.

1984. Larval instar determination and temperature‐development studies of immature stages of the common armyworm, *Mythimna convecta* (Walker) (Lepidoptera: Noctuidae). J. Aust. Entomol. Soc.
23:91–97.

Steigenga, M. J.
, and 
K.
Fischer

2009. Fitness consequences of variation in developmental temperature in a butterfly. Journal of Thermal Biology.
34:244–249.

Stenseng, L.
, 
H.
Skovgard
, and 
P.
Holter

2003. Life table studies of the pupal parasitoid *Urolepis rufipes* (Hymenoptera: Pteromalidae) on the house fly *Musca domestica* (Diptera: Muscidae) in Denmark. Environ. Entomol.
32:717–725.

Stevens, M. M.

1998. Development and survival of *Chironomus tepperi* Skuse (Diptera: Chironomidae) at a range of constant temperatures. Aquat. Insects.
20:181–188.

Stoyenoff, J. L.
, 
J. A.
Witter
, 
M. E.
Montgomery
, and 
C. A.
Chilcote

1994. Effects of host switching on gypsy moth (*Lymantria dispar* (L.)) under field conditions. Oecologia.
97:143–157.2831392310.1007/BF00323144

Strom, B. L.
, and 
F. P.
Hain

1996. Host choice of late instar gypsy moths (Lepidoptera: Lymantriidae) between loblolly pine and sweetgum. Environ. Entomol.
25:603–610.

Syme, P. D.

1972. The influence of constant temperatures on the non‐diapause development of *Hyssopus thymus* (Hymenoptera: Eulophidae). Can. Entomol.
104:113–120.

Tang, J. ‐ J.
, 
H. ‐ M.
He
, 
T.
Geng
, 
S.
Fu
, and 
F. ‐ S.
Xue

2016. Life history responses of the cabbage beetle *Colaphellus bowringi* to temperature change. Entomol. Res.
46:337–344.

Terada, K.
, 
K.
Matsumura
, and 
T.
Miyatake

2019. Effects of temperature during successive generations on life‑history traits in a seed beetle *Callosobruchus chinensis* (Chrysomelidae: Coleoptera). Appl. Entomol. Zool.
54:459–464.

Teulon, D. A. J.
, and 
D. R.
Penman

1991. Effects of temperature and diet on oviposition rate and development time of the New Zealand flower thrips, *Thrips obscuratus*
. Entomol. Exp. Appl.
60:143–155.

Thiery, D.
, and 
J.
Moreau

2005. Relative performance of European grapevine moth (*Lobesia botrana*) on grapes and other hosts. Oecologia.
143:548–557.1579142810.1007/s00442-005-0022-7

Thompson, L. M.
, 
T. M.
Faske
, 
N.
Banahene
, 
D.
Grim
, 
S. J.
Agosta
, 
D.
Parry
, et al. 2017. Variation in growth and developmental responses to supraoptimal temperatures near latitudinal range limits of gypsy moth *Lymantria dispar* (L.), an expanding invasive species. Physiol. Entomol.
42:181–190.

Tian, S.
, 
T.
Gu
, 
C.
Chen
, 
X.
Zhao
, 
P.
Liu
, and 
D.
Hao

2020. The effects of temperature and host size on the development of *Brachymeria lasus* parasitising *Hyphantria cunea*
. J. For. Res.
32:401–407.

Tochen, S.
, 
D. T.
Dalton
, 
N.
Wiman
, 
C.
Hamm
, 
P. W.
Shearer
, and 
V. M.
Walton

2014. Temperature‐related development and population parameters for *Drosophila suzukii* (Diptera: Drosophilidae) on cherry and blueberry. Environ. Entomol.
43:501–510.2461296810.1603/EN13200

Togashi, K.
, and 
J.
Kodani

1990. Effect of temperature on the development of *Ivela auripes* (Butler) (Lepidoptera: Lymantriidae). J. Jpn. For. Soc.
72:316–320.

Tokuda, M.
, and 
M.
Matsumura

2005. Effect of temperature on the development and reproduction of the maize orange leafhopper *Cicadulina bipunctata* (Melichar) (Homoptera: Cicadellidae). Appl. Entomol. Zool.
40:213–220.

Tomberlin, J. K.
, 
P. H.
Adler
, and 
H. M.
Myers

2009. Development of the black soldier fly (Diptera: Stratiomyidae) in relation to temperature. Environ. Entomol.
38:930–934.1950880410.1603/022.038.0347

Toyoshima, S.
, 
T.
Arai
, and 
K.
Yaginuma

2010. Effect of constant temperatures on the development of peach fruit moth, *Carposina sasakii* (Lepidoptera: Carposinidae). Bull. Natl. Inst. Fruit Tree Sci.
10:1–8.

Traore, L.
, 
J. ‐ G.
Pilon
, 
F.
Fournier
, and 
G.
Boivin

2006. Adaptation of the developmental process of *Anaphes victus* (Hymenoptera: Mymaridae) to local climatic conditions across North America. Ann. Entomol. Soc. Am.
99:1121–1126.

Tsukada, M.
, 
M.
Asai
, and 
H.
Higuchi

2005. Developmental period and adult size of *Haptoncus ocularis* (Coleoptera: Nitidulidae) at four temperature conditions. Appl. Entomol. Zool.
40:489–495.

Tsukada, M.
, 
D.
Tanaka
, and 
H.
Higuchi

2008. Thermal requirement for development of *Carpophilus marginellus* (Coleoptera: Nitidulidae), a potential pollinator of cherimoya and atemoya trees (Magnoliales: Annonaceae). Appl. Entomol. Zool.
43:281–285.

Ugine, T. A.
, 
J. P.
Sanderson
, and 
S. P.
Wraight

2007. Developmental times and life tables for shore flies, *Scatella tenuicosta* (Diptera: Ephydridae), at three temperatures. Environ. Entomol.
36:989–997.1828471910.1603/0046-225x(2007)36[989:dtaltf]2.0.co;2

Ullah, M. S.
, and 
U. T.
Lim

2015. Life history characteristics of *Frankliniella occidentalis* and *Frankliniella intonsa* (Thysanoptera: Thripidae) in constant and fluctuating temperatures. J. Econ. Entomol.
108:1000–1009.2647022210.1093/jee/tov035

Urbaneja, A.
, 
R.
Hinarejos
, 
E.
Llacer
, 
A.
Garrido
, and 
J. ‐ A.
Jacas

2002. Effect of temperature on life history of *Cirrospilus vittatus* (Hymenoptera: Eulophidae), an ectoparasitoid of *Phyllocnistis citrella* (Lepidoptera: Gracillariidae). J. Econ. Entomol.
95:250–255.1201999710.1603/0022-0493-95.2.250

Uyi, O. O.
, 
C.
Zachariades
, 
M. P.
Hill
, and 
A. J.
McConnachie

2016. Temperature‐dependent performance and potential distribution of *Pareuchaetes insulata*, a biological control agent of *Chromolaena odorata* in South Africa. BioControl.
61:815–825.

Varikou, K.
, 
I.
Tsitsipis
, 
V.
Alexandrakis
, and 
M.
Hoddle

2009. Effect of temperature on the development and longevity of *Pezothrips kellyanus* (Thysanoptera: Thripidae). Ann. Entomol. Soc. Am.
102:835–841.

Walker, P. W.

2011. Biology and development of *Chaetophthalmus dorsalis* (Malloch) (Diptera: Tachinidae) parasitising *Helicoverpa armigera* (Hübner) and *H. punctigera* Wallengren (Lepidoptera: Noctuidae) larvae in the laboratory. Aust. J. Entomol.
50:309–318.

Wanderley, M. J. A.
, and 
F. S.
Ramalho

1999. Effects of the temperature on the development of *Supputius cincticeps* (Stäl) (Heteroptera: Pentatomidae) fed on *Musca domestica* L. larvae. An. Soc. Ent. Bras.
28:121–129.

Wang, X. G.
, and 
R. H.
Messing

2004. Fitness consequences of body‐size‐dependent host species selection in a generalist ectoparasitoid. Biological Control.
31:227–236.

Wang, L.
, 
P.
Shi
, 
C.
Chen
, and 
F.
Xue

2013. Effect of temperature on the development of *Laodelphax striatellus* (Homoptera: Delphacidae). J. Econ. Entomol.
106:107–114.2344802110.1603/ec12364

Wang, X. ‐ G.
, 
M. A.
Serrato
, 
Y.
Son
, 
V. M.
Walton
, 
B. N.
Hogg
, and 
K. M.
Daane

2018. Thermal performance of two indigenous pupal parasitoids attacking the invasive *Drosophila suzukii* (Diptera: Drosophilidae). Environ. Entomol.
47:764–772.2963536610.1093/ee/nvy053

Weathersbee, A. A.
, 
C. L.
McKenzie
, and 
Y. Q.
Tang

2004. Host plant and temperature effects on *Lysiphlebus testaceipes* (Hymenoptera: Aphidiidae), a native parasitoid of the exotic brown citrus aphid (Homoptera: Aphididae). Ann. Entomol. Soc. Am.
97:476–480.10.1603/0022-0493-97.4.123315384331

Weaver, D. K.
, and 
J. E.
Throne

1994. Life history data for *Sitotroga cerealella* (Olivier) (Lepidoptera: Gelechiidae) in farm‐stored corn and the importance of suboptimal environmental conditions in insect population modelling for bulk commodities. Pp. 599–604 *in* Proceedings of the 6th International Working Conference on Stored‐Product Protection.

Willott, S. J.
, and 
M.
Hassall

1998. Life‐history responses of British grasshoppers (Orthoptera: Acrididae) to temperature change. Funct. Ecol.
12:232–241.

Woodson, W. D.
, and 
J. J.
Jackson

1996. Developmental rate as a function of temperature in northern corn rootworm (Coleoptera: Chrysomelidae). Ann. Entomol. Soc. Am.
89:226–230.

Xia, Q. ‐ W.
, 
C.
Chen
, 
J. ‐ J.
Tang
, 
H. ‐ M.
He
, and 
F. ‐ S.
Xue

2019. A reverse temperature‐size rule associated with a negative relationship between larval development time and pupal weight in a tropical population of *Ostrinia furnacalis*
. Physiol. Entomol.
44:209–214.

Xiao, L.
, 
H. ‐ M.
He
, 
L. ‐ L.
Huang
, 
T.
Geng
, 
S.
Fu
, and 
F. ‐ S.
Xue

2016. Variation of life‐history traits of the Asian corn borer, *Ostrinia furnacalis* in relation to temperature and geographical latitude. Ecol. E.
6:5129–5143.10.1002/ece3.2275PMC498449227551371

Yasuda, H.
, and 
A. F. G.
Dixon

2002. Sexual size dimorphism in the two spot ladybird beetle *Adalia bipunctata*: developmental mechanism and its consequences for mating. Ecol. Entomol.
27:493–498.

Yi, S. ‐ J.
, 
R. J.
Hopkins
, 
X. ‐ Y.
Chen
, 
Z. ‐ M.
Chen
, 
X.
Wang
, and 
G. ‐ H.
Huang

2020. Effects of temperature on the development and fecundity of *Microplitis similis* (Hymenoptera: Braconidae), a parasitoid of *Spodoptera litura* (Lepidoptera: Noctuidae). Physiol. Entomol.
45:95–102.

Zamani, A. A.
, 
A.
Talebi
, 
Y.
Fathipour
, and 
V.
Baniameri

2007. Effect of temperature on life history of *Aphidius colemani* and *Aphidius matricariae* (Hymenoptera: Braconidae), two parasitoids of *Aphis gossypii* and *Myzus persicae* (Homoptera: Aphididae). Environ. Entomol.
36:263–271.1744536010.1603/0046-225X-36.2.263

Zerbino, M. S.
, 
N. A.
Altier
, and 
A. R.
Panizzi

2013. Effect of photoperiod and temperature on nymphal development and adult reproduction of *Piezodorus guildinii* (Heteroptera: Pentatomidae). Fla. Entomol.
96:572–582.

Zhang, S. ‐ C.
, 
F.
Zhu
, 
X. ‐ L.
Zheng
, 
C. ‐ L.
Lei
, and 
X. ‐ M.
Zhou

2012. Survival and developmental characteristics of the predatory bug *Orius similis* (Hemiptera: Anthocoridae) fed on *Tetranychus cinnabarinus* (Acari: Tetranychidae) at three constant temperatures. Eur. J. Entomol.
109:503–508.

## Supporting information

Appendix S1. Phylogenetic relationships of the species included in the meta‐analysis of sex‐specific plasticity in development time.

Appendix S1. Methodology to quantitatively compare sex differences in diet‐ and temperature‐induced development time plasticity

Figure S1. The distribution of individual effect sizes

Supplementary Material. Table S1. Sources of original data, and variables derived from these data

Table S2. Sexual development time dimorphism (SDTD) in major insect orders, based on the species examined.

Table S3. Sources of original data and variables derived from these data: ln‐slope = logarithmically transformed RMA regression slope of male development time on female development time, gradient length = ratio of maximum and minimum development time.
