# Supplementary material for: Limited sex differences in plastic responses suggest evolutionary conservatism of thermal reaction norms: A meta‐analysis in insects
Source: Evol Lett. 2022 Nov 2;6(6):394–411. doi: 10.1002/evl3.299 (PMC9783480; doi:10.1002/evl3.299)

Supporting Information

Appendix S1. Phylogenetic relationships of the species included in the meta-analysis of sex-specific plasticity in development time.

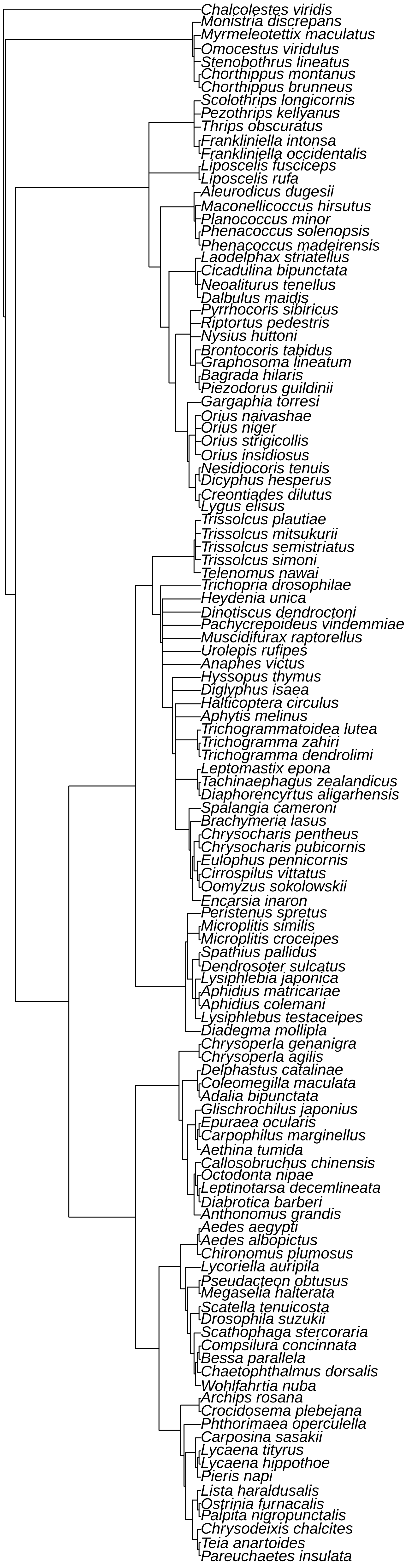

Supplement: Supplementary file 1 — Appendix S1. Phylogenetic relationships of the species included in the meta‐analysis of sex‐specific plasticity in development time. [file EVL3-6-394-s005.pdf]
