# Supplementary material for: Limited sex differences in plastic responses suggest evolutionary conservatism of thermal reaction norms: A meta‐analysis in insects
Source: Evol Lett. 2022 Nov 2;6(6):394–411. doi: 10.1002/evl3.299 (PMC9783480; doi:10.1002/evl3.299)
Supplement: Supplementary file 2 — Appendix S1. Methodology to quantitatively compare sex differences in diet‐ and temperature‐induced development time plasticity [file EVL3-6-394-s006.pdf]

Supporting Information

Appendix S1. Methodology to quantitatively compare sex differences in diet- and temperature-induced development time plasticity

We applied an original permutation test-based meta-analysis to compare sex differences in development time responses to temperature and diet. Such an approach was taken because, to our knowledge, there is no directly applicable meta-analysis technique for this kind of data that would allow the incorporation of measurement variance.

For each species, logarithm-transformed RMA regression slopes of male development times on female development times were estimated to quantify sex differences in development time plasticity (see *Operations with primary data* in the main text, for details), separately for data sets based on temperature/diet manipulations. Variability of each log-slope estimate was accounted for by performing the following steps. A uniform distribution was constructed around each data point (i.e., each treatment average), using its standard error[1] to determine the distribution width. A random point was then drawn from each such distribution and transformed to a log-scale, providing a simulated sample with N equal to the number of treatments in the respective data set.

The obtained sample was used to calculate a simulated log-slope for each data set. This slope was then divided by the respective log-slope variability estimate to avoid the effect of possible systematic differences in the standard errors of the log-slopes of temperature and diet. If several data sets were available for a particular species × environment (i.e. temperature or diet) combination, a separate log-slope was calculated for each of these data sets, and the respective log-slopes were averaged. Species-wise differences between the absolute values of these log-slopes for diet and temperature were calculated and recorded. Finally, these species-wise differences were averaged to produce a test statistic. The process was repeated 1,000 times, and thus 1,000 test statistic values were produced. This distribution is referred to as the observed distribution, and is denoted *D_obs*.

To produce a reference statistic, we used the methodology laid out above, with the exception that, for each species, the difference between the absolute values of the log-slopes was randomly multiplied by 1 or −1 (corresponding to a random permutation of the log-slope of diet and log-slope of temperature). For a fixed permutation pattern, this was carried out 1,000 times, and again, thus 1,000 values of the reference statistic were produced. This distribution is referred to as the null distribution, and is denoted *D_null*.

In total, we generated 1,000 permutation patterns and thus also 1,000 null distributions D_null1, ..., D_null1000. As a next step, we used a one-sided Kolmogorov-Smirnov test to compare *D_obs* with each of the *D_null* distributions. This was done to test the hypothesis that the distribution *D_obs* is stochastically larger than the respective distribution *D_null* (i.e., the cumulative distribution function of *D_obs* is below the respective cumulative distribution function of *D_null*). We refer to each such test as a local test. Thus, 1,000 local tests were carried out. The *P*-value of the global test was calculated as the ratio of significant local tests divided by the total number of local tests performed.

[1] Please note that some primary data sets had sample sizes available but lacked the standard errors of the treatment means. To make use of such data, we assumed that the standard deviation of single observations is proportional to the mean. This implies that the standard error of the treatment mean is proportional to the ratio of the treatment mean and the square root of the sample size. We used this equation to estimate the proportionality constant C

based on the data that had both sample sizes and standard errors available (separately for females/males and temperature/diet; 4 proportionality constants in total), and then used the constant C to estimate the standard error for the data that had only sample sizes available.
