# Supplementary material for: Limited sex differences in plastic responses suggest evolutionary conservatism of thermal reaction norms: A meta‐analysis in insects
Source: Evol Lett. 2022 Nov 2;6(6):394–411. doi: 10.1002/evl3.299 (PMC9783480; doi:10.1002/evl3.299)

Supplementary Material

Figure S1. The distribution of individual effect sizes (logarithmically transformed RMA regression slopes of male development time on female development time = ln-slopes) describing sex differences in a) temperature- and b) diet-induced plasticity in total development time. Note that ln-slopes describing sex-specific responses to temperature are mostly close to zero, corresponding to a small sex difference in development time plasticity. By contrast, ln-slopes quantifying sex differences in diet-induced plasticity are much more widely scattered, indicating that diet-induced reaction norms often substantially differ between the sexes. Data on sex differences in diet-induced plasticity were obtained from a recent study by Teder et al. (2021).

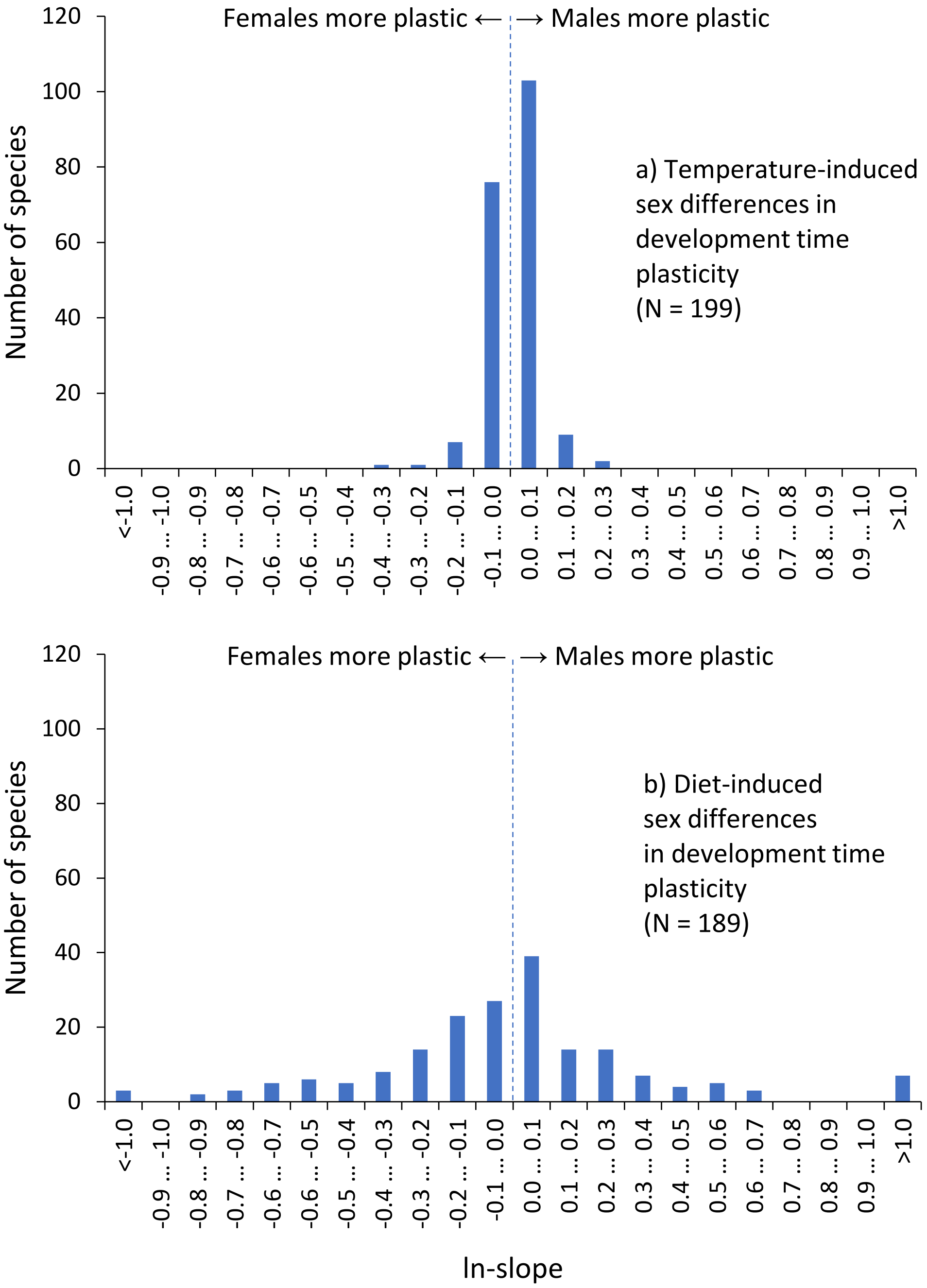

Supplement: Supplementary file 3 — Figure S1. The distribution of individual effect sizes [file EVL3-6-394-s004.pdf]
