# Supplementary material for: Limited sex differences in plastic responses suggest evolutionary conservatism of thermal reaction norms: A meta‐analysis in insects
Source: Evol Lett. 2022 Nov 2;6(6):394–411. doi: 10.1002/evl3.299 (PMC9783480; doi:10.1002/evl3.299)
Supplement: Supplementary file 4 — Supplementary Material. Table S1. Sources of original data, and variables derived from these data [file EVL3-6-394-s002.pdf]

Supplementary Material. Table S1. Sources of original data, and variables derived from these data: logarithmically transformed RMA regression slopes of male development time on female development time (= ln-slopes) and their standard error estimates (= SE of ln-slopes), separately for larval and total development time; cross-treatment correlations between average male and female development times (presented only for studies with at least three treatments); numbers of treatments in source studies; mean sex difference in larval and total development time (= SDTDlarval and SDTDtotal, respectively) averaged across treatments (see main text, for detailed explanations). NA = not available.

ID	Species	Order	Family	ln-slope (larval)	SE of ln- slope (larval)	Correlation (larval)	# treatments (larval)	ln-slope (total)	SE of ln-slope (total)	Correlation (total)	# treatments (total)	Mean $SDTD_{larval}$	Mean $SDTD_{total}$	Source
1	Frankliniella occidentalis	Thysanoptera	Thripidae	0.2134	NA	NA	2	-0.0587	0.0105	NA	2	0.013	0.016	Ullah & Lim 2015, Journal of Economic Entomology 108, 1000-1009
2	Frankliniella intonsa	Thysanoptera	Thripidae	-0.0529	NA	NA	2	-0.0889	0.0173	NA	2	0.034	0.030	Ullah & Lim 2015, Journal of Economic Entomology 108, 1000-1009
3	Trichogramma zahiri	Hymenoptera	Trichogrammatidae	NA	NA	NA	NA	0.0255	0.0011	0.999	5	NA	0.022	Bari et al. 2015, Environmental Entomology 44, 368-378
4	Drosophila suzukii	Diptera	Drosophilidae	-0.0253	0.0064	0.997	5	-0.0108	0.0032	0.999	5	-0.008	-0.015	Tochen et al. 2014, Environmental Entomology 43, 501-510
5	Chorthippus montanus	Orthoptera	Acrididae	0.0058	0.0344	NA	2	0.0058	0.0360	NA	2	0.052	0.052	Rohde et al. 2015, Biological Journal of the Linnean Society 115, 48-57
6	Chorthippus montanus	Orthoptera	Acrididae	-0.1048	0.0366	NA	2	-0.1048	0.0359	NA	2	0.154	0.154	Rohde et al. 2015, Biological Journal of the Linnean Society 115, 48-57
7	Chorthippus montanus	Orthoptera	Acrididae	0.0008	0.0417	NA	2	0.0008	0.0424	NA	2	0.250	0.250	Rohde et al. 2015, Biological Journal of the Linnean Society 115, 48-57
8	Lista haraldusalis	Lepidoptera	Pyralidae	0.0054	0.0025	>0.999	6	0.0071	0.0020	>0.999	6	0.005	0.007	Liu et al. 2014, Journal of Asia-Pacific Entomology 17, 803-810
9	Trichogrammatoidea lutea	Hymenoptera	Trichogrammatidae	NA	NA	NA	NA	0.0122	0.0005	>0.999	5	NA	-0.001	Mawela et al. 2013, Biological Control 64, 211-216
10	Trichogrammatoidea lutea	Hymenoptera	Trichogrammatidae	NA	NA	NA	NA	-0.0014	0.0034	0.998	4	NA	-0.004	Mawela et al. 2013, Biological Control 64, 211-216
11	Trichogrammatoidea lutea	Hymenoptera	Trichogrammatidae	NA	NA	NA	NA	-0.0197	0.0008	>0.999	5	NA	-0.015	Mawela et al. 2013, Biological Control 64, 211-216
12	Phthorimaea operculella	Lepidoptera	Gelechiidae	0.0130	0.0028	>0.999	6	-0.0088	0.0015	>0.999	6	0.011	0.002	Golizadeh & Zalucki 2012, Insect Science 19, 609-620
13	Euscepes postfasciatus	Coleoptera	Curculionidae	-0.0692	0.0408	0.993	4	-0.0514	NA	0.996	4	0.012	-0.001	Shimoji 2011, Applied Entomology & Zoology 46, 1-54
14	Chaetophthalmus dorsalis	Diptera	Tachinidae	-0.0407	0.0077	0.996	5	-0.0503	0.0046	0.997	5	0.077	0.089	Walker 2011, Australian Journal of Entomology 50, 309-318
15	Carposina sasakii	Lepidoptera	Carposinidae	0.0197	0.0029	0.999	7	0.0045	0.0013	>0.999	7	0.031	0.007	Toyoshima et al. 2010, Bulletin of the National Institute of Fruit Tree Science 10, 1-8
16	Octodonta nipae	Coleoptera	Chrysomelidae	NA	NA	NA	NA	0.0221	0.0027	0.999	6	NA	0.045	Hou & Weng 2010, Environmental Entomology 39, 1676-1684
17	Pezothrips kellyanus	Thysanoptera	Thripidae	-0.0281	NA	0.996	5	0.0198	0.0027	0.998	5	-0.002	-0.003	Varikou et al. 2009, Annals of the Entomological Society of America 102, 835-841
18	Hermetia illucens	Diptera	Stratiomyidae	0.1549	0.0582	NA	2	0.1176	NA	NA	2	0.038	0.020	Tomberlin et al. 2009, Environmental Entomology 38, 930-934
19	Creontiades dilutus	Hemiptera	Miridae	-0.0075	0.0020	>0.999	9	-0.0075	0.0020	>0.999	9	-0.018	-0.018	Khan et al. 2009, Australian Journal of Entomology 48, 210-216
20	Aulacaspis yasumatsui	Hemiptera	Diaspididae	-0.2469	0.0166	0.969	4	-0.1061	NA	0.989	4	0.139	0.085	Cave et al. 2009, Florida Entomologist 92, 578-581
21	Carpophilus marginellus	Coleoptera	Nitidulidae	0.0336	0.0042	>0.999	4	0.0031	0.0023	>0.999	4	-0.003	-0.010	Tsukada et al. 2008, Applied Entomology & Zoology 43, 281-285
22	Leptinotarsa decemlineata	Coleoptera	Chrysomelidae	NA	NA	NA	NA	0.0456	0.0061	NA	2	NA	-0.046	Lyytinen et al. 2008, Entomologia Experimentalis et Applicata 127, 157-167
23	Leptinotarsa decemlineata	Coleoptera	Chrysomelidae	NA	NA	NA	NA	-0.0515	0.0047	NA	2	NA	0.005	Lyytinen et al. 2008, Entomologia Experimentalis et Applicata 127, 157-167
24	Leptinotarsa decemlineata	Coleoptera	Chrysomelidae	NA	NA	NA	NA	-0.0078	0.0042	NA	2	NA	-0.024	Lyytinen et al. 2008, Entomologia Experimentalis et Applicata 127, 157-167
25	Trichogramma dendrolimi	Hymenoptera	Trichogrammatidae	NA	NA	NA	NA	0.0007	0.0023	>0.999	5	NA	0.019	Ishijima et al. 2008, Applied Entomology and Zoology 52, 193-200
26	Trichogramma dendrolimi	Hymenoptera	Trichogrammatidae	NA	NA	NA	NA	0.0101	0.0012	>0.999	5	NA	0.011	Ishijima et al. 2008, Applied Entomology and Zoology 52, 193-200
27	Archips rosanus	Lepidoptera	Tortricidae	0.0192	NA	0.998	4	-0.0179	0.0046	0.999	4	0.120	0.104	Doganlar 2008, Journal of Plant Protection Research 48, 63-72
28	Maconellicoccus hirsutus	Hemiptera	Pseudococcidae	0.0678	0.0026	0.985	4	0.0678	0.0027	0.985	4	0.038	0.038	Chong et al. 2008, Environmental Entomology 37, 323-332
29	Scatella tenuicosta	Diptera	Ephydridae	-0.0724	0.0066	>0.999	3	0.0103	0.0044	>0.999	3	0.000	-0.013	Ugine et al. 2007, Environmental Entomology 36, 989-997
30	Aphidius colemani	Hymenoptera	Braconidae	NA	NA	NA	NA	0.0134	0.0169	>0.999	5	NA	0.021	Zamani et al. 2007, Environmental Entomology 36, 263-271
31	Aphidius colemani	Hymenoptera	Braconidae	NA	NA	NA	NA	-0.0249	0.0163	0.999	5	NA	0.038	Zamani et al. 2007, Environmental Entomology 36, 263-271
32	Aphidius matricariae	Hymenoptera	Braconidae	NA	NA	NA	NA	0.0113	0.0131	>0.999	5	NA	0.020	Zamani et al. 2007, Environmental Entomology 36, 263-271
33	Aphidius matricariae	Hymenoptera	Braconidae	NA	NA	NA	NA	-0.0096	0.0148	>0.999	5	NA	0.015	Zamani et al. 2007, Environmental Entomology 36, 263-271
34	Librodor japonicus	Coleoptera	Nitidulidae	0.0312	NA	0.992	5	-0.0236	0.0074	0.995	5	-0.011	-0.026	Okada & Miyatake 2007, Applied Entomology & Zoology 42, 411-417
35	Chrysocharis pubicornis	Hymenoptera	Eulophidae	-0.0412	0.0028	0.998	5	-0.0021	0.0019	0.999	5	-0.009	0.017	Larios et al. 2007, Applied Entomology and Zoology 42, 189-197
36	Lymantria dispar	Lepidoptera	Lymantriidae	0.0425	0.0302	NA	2	NA	NA	NA	2	0.280	NA	Karolewski et al. 2007, Dendrobiology 58, 43-49
37	Compsilura concinnata	Diptera	Tachinidae	NA	NA	NA	NA	0.0512	0.0041	0.997	6	NA	0.088	Ichiki & Nakamura 2007, Japan Agricultural Research Quarterly 41, 227-232
38	Pnigalio pectinicornis	Hymenoptera	Eulophidae	-0.8346	0.1868	0.786	3	-0.3611	0.0444	0.387	3	0.048	0.053	Kalaitzaki et al. 2007, Environmental Entomology 36, 497-505
39	Pnigalio pectinicornis	Hymenoptera	Eulophidae	0.3602	0.0278	>0.999	3	0.2616	0.0090	0.999	3	0.081	0.016	Kalaitzaki et al. 2007, Environmental Entomology 36, 497-505
40	Aethina tumida	Coleoptera	Nitidulidae	0.0109	NA	NA	2	-0.0037	0.0019	NA	2	-0.010	-0.018	Guzman & Frake 2007, Journal of Apicultural Research 46, 88-93
41	Orius similis	Hemiptera	Anthocoridae	-0.0043	0.0085	NA	2	-0.0043	0.0085	NA	2	0.017	0.017	Ahmadi et al. 2007, Turkish Journal of Entomology 31, 253-268
42	Orius similis	Hemiptera	Anthocoridae	0.0034	0.0056	NA	2	0.0034	0.0056	NA	2	0.032	0.032	Ahmadi et al. 2007, Turkish Journal of Entomology 31, 253-268
43	Anaphes victus	Hymenoptera	Mymaridae	NA	NA	NA	NA	0.0062	0.0010	0.998	11	NA	0.057	Traore et al. 2006, Annals of the Entomological Society of America 99, 1121-1126
44	Epiphyas postvittana	Lepidoptera	Tortricidae	0.1835	0.0197	NA	2	0.1544	NA	NA	2	0.115	0.086	Mo et al. 2006, Journal of Economic Entomology 99, 1321-1326
45	Epiphyas postvittana	Lepidoptera	Tortricidae	-0.1053	0.0411	NA	2	0.0131	NA	NA	2	0.113	0.070	Mo et al. 2006, Journal of Economic Entomology 99, 1321-1326
46	Epiphyas postvittana	Lepidoptera	Tortricidae	-0.2580	0.0272	NA	2	-0.1248	NA	NA	2	0.108	0.072	Mo et al. 2006, Journal of Economic Entomology 99, 1321-1326
47	Trissolcus semistriatus	Hymenoptera	Scelionidae	NA	NA	NA	NA	0.0778	0.0022	>0.999	4	NA	0.126	Kivan & Kilic 2006, Entomological Science 9, 39-46
48	Teia anartoides	Lepidoptera	Lymantriidae	-0.0970	0.0046	0.987	4	-0.0305	0.0028	0.997	4	0.365	0.078	Charles et al. 2006, New Zealand Entomologist 29, 27-36
49	Aedes albopictus	Diptera	Culicidae	0.1003	0.0040	NA	2	NA	NA	NA	2	0.106	NA	Armbruster & Conn 2006, Annals of the Entomological Society of America 99, 1234-1243
50	Aedes albopictus	Diptera	Culicidae	0.0291	0.0043	NA	2	NA	NA	NA	2	0.149	NA	Armbruster & Conn 2006, Annals of the Entomological Society of America 99, 1234-1243
51	Aedes albopictus	Diptera	Culicidae	0.0173	0.0057	NA	2	NA	NA	NA	2	0.107	NA	Armbruster & Conn 2006, Annals of the Entomological Society of America 99, 1234-1243
52	Aedes albopictus	Diptera	Culicidae	0.0463	0.0069	NA	2	NA	NA	NA	2	0.152	NA	Armbruster & Conn 2006, Annals of the Entomological Society of America 99, 1234-1243
53	Aedes albopictus	Diptera	Culicidae	0.1084	0.0033	NA	2	NA	NA	NA	2	0.138	NA	Armbruster & Conn 2006, Annals of the Entomological Society of America 99, 1234-1243
54	Aedes albopictus	Diptera	Culicidae	0.0833	0.0044	NA	2	NA	NA	NA	2	0.100	NA	Armbruster & Conn 2006, Annals of the Entomological Society of America 99, 1234-1243
55	Aedes albopictus	Diptera	Culicidae	0.0188	0.0031	NA	2	NA	NA	NA	2	0.158	NA	Armbruster & Conn 2006, Annals of the Entomological Society of America 99, 1234-1243
56	Aedes albopictus	Diptera	Culicidae	0.0719	0.0031	NA	2	NA	NA	NA	2	0.118	NA	Armbruster & Conn 2006, Annals of the Entomological Society of America 99, 1234-1243
57	Aedes albopictus	Diptera	Culicidae	0.0622	0.0032	NA	2	NA	NA	NA	2	0.104	NA	Armbruster & Conn 2006, Annals of the Entomological Society of America 99, 1234-1243
58	Epuraea ocularis	Coleoptera	Nitidulidae	-0.0065	NA	>0.999	4	-0.0008	0.0025	>0.999	4	-0.008	-0.007	Tsukada et al. 2005, Applied Entomology & Zoology 40, 489-495
59	Cicadulina bipunctata	Hemiptera	Cicadellidae	0.0457	0.0030	>0.999	6	0.0457	0.0031	>0.999	6	0.009	0.009	Tokuda & Matsumura 2005, Applied Entomology & Zoology 40, 213-220
60	Adoxophyes honmai	Lepidoptera	Tortricidae	0.0895	0.0019	>0.999	6	0.0571	NA	>0.999	6	0.107	0.054	Nabeta et al. 2005, Applied Entomology & Zoology 40, 231-238
61	Microplitis croceipes	Hymenoptera	Braconidae	-0.0795	0.0043	0.998	6	-0.0780	0.0030	>0.999	6	0.030	0.052	Hoang & Takasu 2005, Applied Entomology & Zoology 40, 679-686
62	Anthonomus grandis grandis	Coleoptera	Curculionidae	NA	NA	NA	NA	0.0856	0.0101	0.999	5	NA	0.162	Greenberg et al. 2005, Insect Science 12, 449-459
63	Pseudacteon obtusus	Diptera	Phoridae	0.7777	0.0590	NA	2	0.1852	0.0221	NA	2	0.075	0.062	Folgarait et al. 2005, Environmental Entomology 34, 308-316
64	Pseudacteon obtusus	Diptera	Phoridae	-0.1528	0.0416	NA	2	-0.1852	0.0213	NA	2	-0.110	-0.049	Folgarait et al. 2005, Environmental Entomology 34, 308-316
65	Aricia agestis	Lepidoptera	Lycaenidae	0.0280	0.0081	0.998	3	NA	NA	NA	3	0.028	NA	Burke et al. 2005, Ecological Entomology 30, 613-619
66	Aricia agestis	Lepidoptera	Lycaenidae	-0.0259	0.0082	>0.999	3	NA	NA	NA	3	0.017	NA	Burke et al. 2005, Ecological Entomology 30, 613-619
67	Aricia agestis	Lepidoptera	Lycaenidae	0.0162	0.0151	>0.999	3	NA	NA	NA	3	0.078	NA	Burke et al. 2005, Ecological Entomology 30, 613-619
68	Lysiphlebus testaceipes	Hymenoptera	Aphidiidae	NA	NA	NA	NA	-0.0142	0.0030	>0.999	4	NA	0.006	Weathersbee et al. 2004, Annals of the Entomological Society of America 97, 476-480
69	Dicyphus hesperus	Hemiptera	Miridae	-0.0275	0.0035	0.997	5	-0.0275	0.0033	0.997	5	-0.045	-0.045	Gillespie et al. 2004, Canadian Entomologist 136, 675-683
70	Dicyphus hesperus	Hemiptera	Miridae	0.0998	0.0028	>0.999	5	0.0998	0.0028	>0.999	5	0.026	0.026	Gillespie et al. 2004, Canadian Entomologist 136, 675-683
71	Gargaphia torresi	Hemiptera	Tingidae	-0.0422	0.0156	0.998	5	-0.0403	0.0106	0.999	5	-0.031	-0.018	Silva 2004, Revista Brasileira de Entomologia 48, 547-552
72	Lygus elisus	Hemiptera	Lygaeidae	-0.0143	0.0042	>0.999	6	-0.0143	0.0042	>0.999	6	0.005	0.005	Bommireddy et al. 2004, Environmental Entomology 33, 1549-1553
73	Urolepis rufipes	Hymenoptera	Pteromalidae	NA	NA	NA	NA	-0.0378	0.0090	>0.999	4	NA	0.088	Stenseng et al. 2003, Environmental Entomology 32, 717-725
74	Oomyzus sokolowskii	Hymenoptera	Eulophidae	NA	NA	NA	NA	-0.0062	0.0027	>0.999	3	NA	0.015	Mahmood et al. 2003, Bulletin of Entomological Research 93, 169-177
75	Oomyzus sokolowskii	Hymenoptera	Eulophidae	NA	NA	NA	NA	-0.0104	0.0031	>0.999	3	NA	0.011	Mahmood et al. 2003, Bulletin of Entomological Research 93, 169-177
76	Bessa parallela	Diptera	Tachinidae	NA	NA	NA	NA	0.0996	0.0150	NA	2	NA	0.106	Ichiki et al. 2003, Applied Entomology and Zoology 38, 435-439
77	Nysius huttoni	Hemiptera	Lygaeidae	-0.0356	0.0040	0.997	3	-0.0356	0.0039	0.997	3	0.051	0.051	He et al. 2003, Journal of Economic Entomology 96, 1119-1125
78	Tachinaephagus zealandicus	Hymenoptera	Encyrtidae	NA	NA	NA	NA	-0.0036	0.0008	>0.999	3	NA	0.001	Geden et al. 2003, Environmental Entomology 32, 1139-1145
79	Tachinaephagus zealandicus	Hymenoptera	Encyrtidae	NA	NA	NA	NA	-0.0042	0.0008	>0.999	3	NA	0.002	Geden et al. 2003, Environmental Entomology 32, 1139-1145
80	Bicyclus anynana	Lepidoptera	Nymphalidae	-0.0265	0.0045	NA	2	-0.0171	NA	NA	2	0.123	0.080	Fischer et al. 2003, Ecology 84, 3138-3147
81	Bicyclus anynana	Lepidoptera	Nymphalidae	-0.0014	0.0055	NA	2	0.0057	NA	NA	2	0.092	0.066	Fischer et al. 2003, Ecology 84, 3138-3147
82	Chalcolestes viridis	Odonata	Lestidae	0.0285	0.0053	0.998	3	0.0285	0.0053	0.998	3	0.014	0.014	De Block & Stoks 2003, Journal of Evolutionary Biology 16, 986-995
83	Chalcolestes viridis	Odonata	Lestidae	0.0329	0.0051	>0.999	3	0.0329	0.0050	>0.999	3	0.022	0.022	De Block & Stoks 2003, Journal of Evolutionary Biology 16, 986-995
84	Phenacoccus madeirensis	Hemiptera	Pseudococcidae	0.0394	0.0018	>0.999	3	0.0394	0.0017	>0.999	3	-0.111	-0.111	Chong et al. 2003, Annals of the Entomological Society of America 96, 539-543
85	Meteorus gyrator	Hymenoptera	Braconidae	0.0370	0.0059	>0.999	5	0.0330	NA	>0.999	5	0.042	0.051	Bell et al. 2003, Journal of Applied Entomology 127, 332-339
86	Diglyphus isaea	Hymenoptera	Eulophidae	0.0374	0.0011	>0.999	4	0.0354	0.0007	0.999	4	0.036	0.032	Bazzocchi et al. 2003, Biological Control 26, 74-82
87	Diglyphus isaea	Hymenoptera	Eulophidae	0.0174	0.0008	0.998	4	0.0261	0.0007	0.998	4	0.040	0.050	Bazzocchi et al. 2003, Biological Control 26, 74-82
88	Adalia bipunctata	Coleoptera	Coccinellidae	NA	NA	NA	NA	-0.0621	0.0072	NA	2	NA	0.012	Yasuda & Dixon 2002, Ecological Entomology 27, 493-498
89	Cirrospilus vittatus	Hymenoptera	Eulophidae	-0.0435	0.0071	0.995	5	-0.0123	0.0057	>0.999	5	0.015	0.013	Urbaneja et al. 2002, Journal of Economic Entomology 95, 250-255
90	Lycaena hippothoe	Lepidoptera	Lycaenidae	-0.0052	0.0036	>0.999	4	-0.0058	0.0024	>0.999	4	0.110	0.075	Fischer & Fiedler 2002, Evolutionary Ecology 16, 333-349
91	Lycaena hippothoe	Lepidoptera	Lycaenidae	-0.0140	0.0033	0.994	4	0.0053	0.0021	0.997	4	0.126	0.085	Fischer & Fiedler 2002, Evolutionary Ecology 16, 333-349
92	Tachinaephagus zealandicus	Hymenoptera	Encyrtidae	NA	NA	NA	NA	0.0010	0.0005	>0.999	5	NA	0.001	Ferreira de Almeida et al. 2002, Environmental Entomology 31, 375-380
93	Spodoptera cosmioides	Lepidoptera	Noctuidae	NA	NA	NA	NA	0.0130	NA	>0.999	6	NA	-0.055	Bavaresco et al. 2002, Neotropical Entomology 31, 49-54
94	Trissolcus mitsukurii	Hymenoptera	Scelionidae	NA	NA	NA	NA	-0.0129	0.0127	>0.999	5	NA	0.077	Arakawa & Namura 2002, Entomological Science 5, 215-218

ID	Species	Order	Family	ln-slope (larval)	SE of ln- slope (larval)	Correlation (larval)	# treatments (larval)	ln-slope (total)	SE of ln-slope (total)	Correlation (total)	# treatments (total)	Mean $SDTD_{larval}$	Mean $SDTD_{total}$	Source
95	Trissolcus plautiae	Hymenoptera	Scelionidae	NA	NA	NA	NA	0.0188	0.0318	0.853	5	NA	0.127	Arakawa & Namura 2002, Entomological Science 5, 215-218
96	Trissolcus itoi	Hymenoptera	Scelionidae	NA	NA	NA	NA	0.0524	0.0165	>0.999	5	NA	0.067	Arakawa & Namura 2002, Entomological Science 5, 215-218
97	Muscidifurax raptorellus	Hymenoptera	Pteromalidae	NA	NA	NA	NA	-0.0181	0.0032	>0.999	5	NA	0.059	Lysyk 2001, Environmental Entomology 30, 982-992
98	Adoxophyes orana	Lepidoptera	Tortricidae	-0.0785	0.0078	0.996	5	NA	NA	NA	5	0.058	NA	Milonas & Savopoulou-Soultani 2000, Annals of the Entomological Society of America 93, 96-102
99	Lycaena tityrus	Lepidoptera	Lycaenidae	0.1072	0.0080	0.998	3	0.0923	0.0046	0.998	3	0.126	0.096	Fischer & Fiedler 2000, Oikos 90, 372-380
100	Supputius cincticeps	Hemiptera	Pentatomidae	0.0588	0.0056	0.999	6	0.0482	0.0048	0.999	6	0.074	0.060	Wanderley & Ramalho 1999, Anais da Sociedade Entomológica do Brasil 28, 121-129
101	Chorthippus brunneus	Orthoptera	Acrididae	0.0375	0.0078	>0.999	3	0.0375	0.0080	>0.999	3	0.039	0.039	Willott & Hassall 1998, Functional Ecology 12, 232-241
102	Myrmeleotettix maculatus	Orthoptera	Acrididae	0.1757	0.0162	NA	2	0.1757	0.0169	NA	2	0.005	0.005	Willott & Hassall 1998, Functional Ecology 12, 232-241
103	Omocestus viridulus	Orthoptera	Acrididae	0.0983	0.0117	>0.999	3	0.0983	0.0120	>0.999	3	0.039	0.039	Willott & Hassall 1998, Functional Ecology 12, 232-241
104	Stenobothrus lineatus	Orthoptera	Acrididae	0.0909	0.0304	NA	2	0.0909	0.0306	NA	2	0.003	0.003	Willott & Hassall 1998, Functional Ecology 12, 232-241
105	Scathophaga stercoraria	Diptera	Scatophagidae	NA	NA	NA	NA	-0.0448	0.0012	0.999	4	NA	-0.066	Blanckenhorn 1997, Oecologia 111, 318-324
106	Scathophaga stercoraria	Diptera	Scatophagidae	NA	NA	NA	NA	0.0382	0.0028	0.995	7	NA	-0.080	Blanckenhorn 1997, Oecologia 111, 318-324
107	Eristalis arbustorum	Diptera	Syrphidae	0.0803	0.0098	0.998	6	-0.0065	NA	>0.999	6	0.009	0.012	Ottenheim et al. 1996, Heredity 77, 493-499
108	Delia antiqua	Diptera	Anthomyiidae	0.0287	0.0045	0.98	4	0.1104	NA	0.99>0.999	4	0.034	0.053	McDonald & Borden 1995, Annals of the Entomological Society of America 88, 756-763
109	Bicyclus anynana	Lepidoptera	Nymphalidae	0.0523	0.0126	0.988	3	NA	NA	NA	3	0.084	NA	Brakefield & Mazzotta 1995, Journal of Evolutionary Biology 8, 559-573
110	Wohlfahrtia nuba	Diptera	Sarcophagidae	0.0666	0.0031	0.99	5	-0.0083	0.0013	0.998	5	0.058	0.034	Amoudi 1993, Journal of the Egyptian Society of Parasitology 23, 697-705
111	Autographa gamma	Lepidoptera	Noctuidae	-0.0179	0.0026	0.999	5	-0.0058	NA	0.999	5	-0.012	-0.027	Hill & Gatehouse 1992, Bulletin of Entomological Research 82, 335-341
112	Crocidosema plebejana	Lepidoptera	Tortricidae	NA	NA	NA	NA	0.0092	0.0009	>0.999	8	NA	0.040	Hamilton & Zalucki 1991, Australian Journal of Zoology 39, 191-200
113	Ivela auripes	Lepidoptera	Lymantriidae	-0.0117	0.0022	>0.999	3	-0.0061	NA	>0.999	3	0.070	0.071	Togashi & Kodani 1990, Journal of Japanese Forestry Society 72, 316-320
114	Homona magnanima	Lepidoptera	Tortricidae	0.0619	0.0031	0.999	6	0.0336	NA	>0.999	6	0.101	0.037	Mao & Kunimi 1990, Applied Entomology & Zoology 34, 127-130
115	Pieris napi	Lepidoptera	Pieridae	NA	NA	NA	NA	0.0280	0.0016	>0.999	3	NA	0.017	Forsberg & Wiklund 1988, Functional Ecology 2, 81-88
116	Mythimna convecta	Lepidoptera	Noctuidae	0.0575	NA	0.999	5	0.0450	NA	>0.999	5	-0.022	-0.026	Smith 1984, Journal of Australian Entomological Society 23, 91-97
117	Merophyas divulsana	Lepidoptera	Tortricidae	0.0479	0.0156	0.999	7	NA	NA	NA	7	0.041	NA	Allsopp et al. 1983, Journal of Australian Entomological Society 22, 287-291
118	Spodoptera frugiperda	Lepidoptera	Noctuidae	-0.0033	NA	>0.999	3	0.0297	NA	>0.999	3	0.006	-0.044	Combs & Valerio 1980, Environmental Entomology 9, 393-396
119	Spodoptera frugiperda	Lepidoptera	Noctuidae	0.0097	NA	>0.999	3	-0.0321	NA	>0.999	3	-0.037	-0.072	Combs & Valerio 1980, Environmental Entomology 9, 393-396
120	Spodoptera frugiperda	Lepidoptera	Noctuidae	-0.0094	NA	>0.999	3	0.0154	NA	>0.999	3	0.014	-0.032	Combs & Valerio 1980, Environmental Entomology 9, 393-396
121	Spodoptera frugiperda	Lepidoptera	Noctuidae	0.0209	NA	>0.999	3	0.0186	NA	>0.999	3	-0.037	-0.061	Combs & Valerio 1980, Environmental Entomology 9, 393-396
122	Sarcophaga peregrina	Diptera	Sarcophagidae	NA	NA	NA	NA	-0.0068	NA	>0.999	6	NA	-0.002	Buei et al. 1978, Japanese Journal of Sanitary Zoology 29, 125-132
123	Sarcophaga similis	Diptera	Sarcophagidae	NA	NA	NA	NA	0.0036	NA	>0.999	6	NA	-0.030	Buei et al. 1978, Japanese Journal of Sanitary Zoology 29, 125-132
124	Sarcophaga crassipalpis	Diptera	Sarcophagidae	NA	NA	NA	NA	0.0190	NA	0.999	6	NA	0.007	Buei et al. 1978, Japanese Journal of Sanitary Zoology 29, 125-132
125	Monistria discrepans	Orthoptera	Pyrgomorphidae	0.2329	0.0050	0.969	5	0.2329	0.0052	0.969	5	0.213	0.213	Allsopp 1977, Journal of Australian Entomological Society 16, 207-213
126	Hyssopus thymus	Hymenoptera	Eulophidae	NA	NA	NA	NA	-0.0117	0.0031	0.999	5	NA	0.083	Syme 1972, Canadian Entomologist 104, 113-120
127	Plutella xylostella	Lepidoptera	Plutellidae	-0.0258	NA	NA	2	NA	NA	NA	2	0.033	NA	Atwal 1955, Australian Journal of Zoology 3, 185-221
128	Plutella xylostella	Lepidoptera	Plutellidae	0.0431	NA	NA	2	NA	NA	NA	2	0.033	NA	Atwal 1955, Australian Journal of Zoology 3, 185-221
129	Plutella xylostella	Lepidoptera	Plutellidae	-0.0400	NA	NA	2	NA	NA	NA	2	0.062	NA	Atwal 1955, Australian Journal of Zoology 3, 185-221
130	Eutettix tenellus	Hemiptera	Cicadellidae	0.0260	0.0012	0.999	6	0.0260	0.0012	0.999	6	0.045	0.045	Harries & Douglass 1948, Ecological Monographs 18, 45-79
131	Peristenus spretus	Hymenoptera	Braconidae	-0.0452	0.0031	>0.999	4	0.0069	0.0012	>0.999	4	0.042	0.088	Luo et al. 2015, Biocontrol Science and Technology 25, 1410-1425
132	Laodelphax striatellus	Hemiptera	Delphacidae	0.0097	0.0024	0.993	8	0.0097	0.0025	0.993	8	0.054	0.054	Wang et al. 2013, Journal of Economic Entomology 106, 107-114
133	Piezodorus guildinii	Hemiptera	Pentatomidae	-0.0323	0.0101	NA	2	-0.0323	0.0097	NA	2	-0.016	-0.016	Zerbino et al. 2013, Florida Entomologist 96, 572-582
134	Chrysoperla agilis	Neuroptera	Chrysopidae	-0.0339	NA	>0.999	7	-0.0483	0.0080	0.999	7	0.047	0.046	Pappas et al. 2013, Biological Control 64, 291-298
135	Lycoriella auripila	Diptera	Sciaridae	NA	NA	NA	NA	0.0584	0.0013	0.998	7	NA	0.068	Farsani et al. 2013, Journal of Economic Entomology 106, 115-123
136	Lycoriella auripila	Diptera	Sciaridae	NA	NA	NA	NA	0.1199	0.0018	0.989	7	NA	0.113	Farsani et al. 2013, Journal of Economic Entomology 106, 115-123
137	Orius similis	Hemiptera	Anthocoridae	-0.1677	0.0182	0.974	3	-0.1677	0.0181	0.974	3	0.061	0.061	Zhang et al. 2012, European Journal of Entomology 109, 503-508
138	Phenacoccus solenopsis	Hemiptera	Pseudococcidae	-0.0119	0.0030	>0.999	5	-0.0119	0.0028	>0.999	5	-0.029	-0.029	Prasad et al. 2012, Crop Protection 39, 81-88
139	Planococcus minor	Hemiptera	Pseudococcidae	0.0358	0.0034	0.999	3	0.0358	0.0033	0.999	3	-0.047	-0.047	Francis et al. 2012, Florida Entomologist 95, 297-303
140	Orius thripoborus	Hemiptera	Anthocoridae	0.0147	0.0032	>0.999	6	0.0082	0.0020	>0.999	6	-0.024	-0.018	Bonte et al. 2012, Environmental Entomology 41, 989-996
141	Orius naivashae	Hemiptera	Anthocoridae	0.0107	0.0027	0.999	7	0.0225	0.0021	0.999	7	0.012	0.014	Bonte et al. 2012, Environmental Entomology 41, 989-996
142	Chrysoperla genanigra	Neuroptera	Chrysopidae	-0.0037	NA	>0.999	5	-0.0010	0.0022	0.999	5	0.048	0.035	Bezerra et al. 2012, Biological Control 60, 113-118
143	Scolothrips longicornis	Thysanoptera	Thripidae	-0.0634	NA	0.996	6	-0.0204	0.0023	0.999	6	0.288	0.057	Pakyari et al. 2011, Journal of Pest Science 84, 153-163
144	Paracoccus burnerae	Hemiptera	Pseudococcidae	0.0874	NA	0.992	5	0.0874	NA	0.992	5	-0.014	-0.014	Johnson & Giliomee 2011, African Entomology 19, 641-649
145	Palpita nigropunctalis	Lepidoptera	Crambidae	0.0334	0.0025	0.999	6	0.0204	0.0014	>0.999	6	0.011	0.000	Gotoh et al. 2011, Journal of Asia-Pacific Entomology 14, 173-178
146	Liposcelis rufa	Psocoptera	Liposcelididae	0.1716	0.0088	0.978	8	0.1473	0.0064	0.989	8	0.306	0.182	Gautam et al. 2010, Journal of Economic Entomology 103, 1920-1928
147	Leptomastix epona	Hymenoptera	Encyrtidae	NA	NA	NA	NA	-0.1041	0.0101	>0.999	3	NA	0.042	Karamaouna & Copland 2009, BioControl 54, 65-76
148	Delphastus catalinae	Coleoptera	Coccinellidae	-0.2204	NA	0.989	3	-0.0595	0.0070	>0.999	3	-0.010	-0.011	Legaspi et al. 2008, Journal of Insect Science 8:07
149	Telenomus nawai	Hymenoptera	Scelionidae	NA	NA	NA	NA	-0.0258	0.0011	>0.999	4	NA	0.014	Fukuda et al. 2007, Bulletin of Entomological Research 97, 185-190
150	Podisus maculiventris	Hemiptera	Pentatomidae	0.0052	NA	0.999	4	0.0126	NA	>0.999	4	0.040	0.036	Legaspi & Legaspi 2005, Environmental Entomology 34, 990-998
151	Lydella jalisco	Diptera	Tachinidae	0.0470	0.0129	>0.999	3	0.0138	NA	>0.999	3	0.143	0.091	Lauziere et al. 2002, Environmental Entomology 31, 432-437
152	Scirtothrips perseae	Thysanoptera	Thripidae	0.0429	NA	>0.999	5	0.0429	NA	>0.999	5	0.007	0.007	Hoddle 2002, Bulletin of Entomological Research 92, 279-285
153	Orius strigicollis	Hemiptera	Anthocoridae	-0.0335	0.0028	>0.999	4	-0.0335	0.0029	>0.999	4	-0.006	-0.006	Ohta 2001, Applied Entomology & Zoology 36, 483-488
154	Chironomus tepperi	Diptera	Chironomidae	0.0811	NA	0.993	10	0.0629	NA	0.995	10	0.147	0.114	Stevens 1998, Aquatic Insects 20, 181-188
155	Diabrotica barberi	Coleoptera	Chrysomelidae	-0.0118	NA	0.999	7	-0.0164	0.0025	0.999	7	0.021	0.017	Woodson & Jackson 1996, Annals of the Entomological Society of America 89, 226-230
156	Sitotroga cerealella	Lepidoptera	Gelechiidae	NA	NA	NA	NA	-0.0650	NA	0.9>0.9993	3	NA	-0.050	Weaver & Throne 1994, Proceedings of the 6th International Conference on Stored-product Protection 1, 599-604
157	Sitotroga cerealella	Lepidoptera	Gelechiidae	NA	NA	NA	NA	-0.2075	NA	0.973	4	NA	-0.067	Weaver & Throne 1994, Proceedings of the 6th International Conference on Stored-product Protection 1, 599-604
158	Dendrosoter sulcatus	Hymenoptera	Braconidae	NA	NA	NA	NA	-0.0095	0.0029	>0.999	3	NA	0.124	Jones & Stephen 1994, Environmental Entomology 23, 457-463
159	Spathius pallidus	Hymenoptera	Braconidae	NA	NA	NA	NA	0.0120	0.0055	>0.999	4	NA	0.068	Jones & Stephen 1994, Environmental Entomology 23, 457-463
160	Dinotiscus dendroctoni	Hymenoptera	Pteromalidae	NA	NA	NA	NA	0.0120	0.0073	0.999	4	NA	0.056	Jones & Stephen 1994, Environmental Entomology 23, 457-463
161	Heydenia unica	Hymenoptera	Pteromalidae	NA	NA	NA	NA	0.1327	0.0579	NA	2	NA	-0.014	Jones & Stephen 1994, Environmental Entomology 23, 457-463
162	Nosopsyllus laeviceps	Siphonaptera	Ceratophyllidae	NA	NA	NA	NA	0.0118	NA	0.995	5	NA	-0.095	Amin et al. 1993, Journal of Parasitology 79, 193-197
163	Aphytis melinus	Hymenoptera	Aphelinidae	NA	NA	NA	NA	-0.0173	0.0021	>0.999	3	NA	0.014	Abdelrahman 1974, Australian Journal of Zoology 22, 213-230
164	Xenopsylla conformis	Siphonaptera	Xenopsyllidae	0.0673	0.0617	NA	2	NA	NA	NA	2	-0.120	NA	Krasnov et al. 2001, Medical and Veterinary Entomology 15, 249-258
165	Scathophaga stercoraria	Diptera	Scathophagidae	NA	NA	NA	NA	0.0661	NA	>0.999	3	NA	-0.074	Blanckenhorn 1997, Oecologia 109, 342-352
166	Scathophaga stercoraria	Diptera	Scathophagidae	NA	NA	NA	NA	0.0116	NA	>0.999	3	NA	-0.092	Blanckenhorn 1997, Oecologia 109, 342-352
167	Chironomus crassicaudatus	Diptera	Chironomidae	-0.0180	NA	0.976	5	0.0086	0.0118	0.99>0.999	5	0.212	0.172	Frouz et al. 2002, Journal of Economic Entomology 95, 699-705
168	Orgyia vetusta	Lepidoptera	Lymantriidae	0.0030	0.0027	0.999	6	NA	NA	NA	6	0.173	NA	Graeve 2008, Master's thesis, San Jose State University
169	Manduca sexta	Lepidoptera	Sphingidae	0.1856	NA	NA	2	NA	NA	NA	2	0.009	NA	Kingsolver et al. 2009, Evolution 63, 537-541
170	Manduca sexta	Lepidoptera	Sphingidae	-0.3638	NA	NA	2	NA	NA	NA	2	0.007	NA	Kingsolver et al. 2009, Evolution 63, 537-541
171	Lymantria dispar	Lepidoptera	Lymantriidae	0.0803	NA	0.998	3	NA	NA	NA	3	0.102	NA	Lindroth et al. 1997, Physiological Entomology 22, 55-64
172	Lymantria dispar	Lepidoptera	Lymantriidae	0.0378	NA	>0.999	3	NA	NA	NA	3	0.114	NA	Lindroth et al. 1997, Physiological Entomology 22, 55-64
173	Helicoverpa armigera	Lepidoptera	Noctuidae	-0.0116	NA	0.999	3	NA	NA	NA	3	0.021	NA	Chen et al. 2014, Journal of Insect Science 14:19
174	Lycaena hippothoe	Lepidoptera	Lycaenidae	NA	NA	NA	NA	-0.0264	NA	>0.999	4	NA	0.044	Fischer & Fiedler 2001, Journal of Evolutionary Biology 14, 210-218
175	Pyrrhocoris sibiricus	Lepidoptera	Pyrrhocoridae	0.0205	0.0027	>0.999	3	0.0205	0.0027	>0.999	3	-0.016	-0.016	Sakashita et al. 1997, Applied Entomology and Zoology 32, 153-157
176	Pyrrhocoris sibiricus	Lepidoptera	Pyrrhocoridae	0.0249	0.0058	>0.999	3	0.0249	0.0059	>0.999	3	-0.022	-0.022	Sakashita et al. 1997, Applied Entomology and Zoology 32, 153-157
177	Colaphellus bowringi	Coleoptera	Chrysomelidae	-0.0716	0.0009	0.79>0.999	6	-0.0018	NA	0.895	6	0.145	0.100	Tang et al. 2016, Entomological Research 46, 337-344
178	Manduca sexta	Lepidoptera	Sphingidae	0.0423	0.0069	>0.999	3	NA	NA	NA	3	0.047	NA	Diamond & Kingsolver 2010, American Naturalist 175, 1-10
179	Manduca sexta	Lepidoptera	Sphingidae	0.0061	0.0114	>0.999	3	NA	NA	NA	3	0.036	NA	Diamond & Kingsolver 2010, American Naturalist 175, 1-10
180	Hyphantria cunea	Lepidoptera	Erebidae	0.1089	NA	0.998	5	NA	NA	NA	5	0.105	NA	Gomi 2006, Applied Entomology & Zoology 41, 303-307
181	Cabera exanthemata	Lepidoptera	Geometridae	0.0061	0.0049	NA	2	NA	NA	NA	2	0.010	NA	Kivelä et al. 2012, Journal of Evolutionary Biology 25, 881-891
182	Scathophaga stercoraria	Diptera	Scathophagidae	NA	NA	NA	NA	-0.0262	0.0026	0.999	6	NA	-0.074	Blanckenhorn & Henseler 2005, Entomologia Experimentalis et Applicata 116, 159-165
183	Pareuchaetes insulata	Lepidoptera	Erebidae	-0.0328	0.0053	0.998	4	0.0162	0.0033	>0.999	4	0.030	-0.023	Uyi et al. 2016, BioControl 61, 815-825
184	Ostrinia furnacalis	Lepidoptera	Crambidae	0.0306	0.0031	0.995	7	0.0161	0.0025	0.997	7	0.064	0.037	Xiao et al. 2016, Ecology and Evolution 6, 5129-5143
185	Ostrinia furnacalis	Lepidoptera	Crambidae	-0.0319	0.0028	0.997	7	-0.0303	0.0023	0.998	7	0.059	0.036	Xiao et al. 2016, Ecology and Evolution 6, 5129-5143
186	Ostrinia furnacalis	Lepidoptera	Crambidae	-0.0931	0.0028	0.995	7	-0.0742	0.0023	0.996	7	0.110	0.074	Xiao et al. 2016, Ecology and Evolution 6, 5129-5143
187	Ostrinia furnacalis	Lepidoptera	Crambidae	-0.0009	0.0031	0.997	7	-0.0024	0.0025	0.999	7	0.044	0.017	Xiao et al. 2016, Ecology and Evolution 6, 5129-5143
188	Dalbulus maidis	Hemiptera	Cicadellidae	-0.0148	0.0048	0.998	5	-0.0148	0.0047	0.998	5	-0.005	-0.005	Nieuwenhove et al. 2016, Agricultural and Forest Entomology 18, 1-10
189	Diomea cremata	Lepidoptera	Noctuidae	-0.0151	0.0027	>0.999	5	0.0029	NA	>0.999	5	0.011	-0.011	Kitajima et al. 2016, Applied Entomology and Zoology 60, 205-209
190	Megaselia halterata	Diptera	Phoridae	NA	NA	NA	NA	0.0168	0.0010	>0.999	6	NA	0.002	Barzegar et al. 2016, Neotropical Entomology 45, 507-517
191	Megaselia halterata	Diptera	Phoridae	NA	NA	NA	NA	-0.0038	0.0009	>0.999	6	NA	0.013	Barzegar et al. 2016, Neotropical Entomology 45, 507-517
192	Chrysocharis pentheus	Hymenoptera	Eulophidae	NA	NA	NA	NA	0.1895	0.0400	0.985	4	NA	0.067	Mafi & Ohbayashi 2010, Journal of Agricultural Science and Technology 12, 145-154
193	Coleomegilla maculata	Coleoptera	Coccinellidae	NA	NA	NA	NA	0.0220	0.0061	0.999	5	NA	0.004	Morales-Ramos & Rojas 2017, Journal of Insect Science 17, 55

ID	Species	Order	Family	ln-slope (larval)	SE of ln- slope (larval)	Correlation (larval)	# treatments (larval)	ln-slope (total)	SE of ln-slope (total)	Correlation (total)	# treatments (total)	Mean $SDTD_{larval}$	Mean $SDTD_{total}$	Source
194	Halticoptera circulus	Hymenoptera	Pteromalidae	NA	NA	NA	NA	0.0294	0.0026	>0.999	3	NA	0.049	Kemmochi et al. 2016, Bulletin of Entomological Research 106, 322-327
195	Halticoptera circulus	Hymenoptera	Pteromalidae	NA	NA	NA	NA	0.0080	0.0024	>0.999	3	NA	0.029	Kemmochi et al. 2016, Bulletin of Entomological Research 106, 322-327
196	Encarsia inaron	Hymenoptera	Aphelinidae	NA	NA	NA	NA	0.0282	0.0042	0.996	4	NA	0.078	Malekmohammadi et al. 2012, Crop Protection 34, 1-5
197	Liposcelis fusciceps	Psocoptera	Liposcelididae	-0.0122	0.0097	0.986	6	0.0026	0.0070	0.993	6	0.320	0.141	Gautam et al. 2016, Environmental Entomology 45, 237-244
198	Spalangia cameroni	Hymenoptera	Pteromalidae	NA	NA	NA	NA	0.0244	0.0026	>0.999	4	NA	0.064	Skovgard & Nachman 2016, Environmental Entomology 45, 1063-1075
199	Bagrada hilaris	Hemiptera	Pentatomidae	-0.0718	0.0058	0.985	7	-0.0718	0.0059	0.985	7	-0.001	-0.001	Reed et al. 2017, Journal of Economic Entomology 110, 2497-2503
200	Lymantria dispar	Lepidoptera	Lymantriidae	-0.3331	0.0664	NA	2	NA	NA	NA	2	0.187	NA	Thompson et al. 2017, Physiological Entomology 42, 181-190
201	Lymantria dispar	Lepidoptera	Lymantriidae	-4.3497	NA	NA	2	NA	NA	NA	2	0.202	NA	Thompson et al. 2017, Physiological Entomology 42, 181-190
202	Lymantria dispar	Lepidoptera	Lymantriidae	-0.0983	0.0364	NA	2	NA	NA	NA	2	0.180	NA	Thompson et al. 2017, Physiological Entomology 42, 181-190
203	Thrips obscuratus	Thysanoptera	Thripidae	NA	NA	NA	NA	-0.0442	0.0056	0.98>0.999	6	NA	0.096	Teulon & Penman 1991, Entomologia Experimentalis et Applicata 60, 143-155
204	Lysiphebia mirzai	Hymenoptera	Aphidiidae	NA	NA	NA	NA	0.0219	0.0019	>0.999	5	NA	0.010	Liu & Tsai 2002, Environmental Entomology 31, 418-424
205	Ruspolia differens	Orthoptera	Tettigoniidae	-0.0616	NA	0.958	6	-0.0616	NA	0.958	6	0.013	0.013	Lehtovaara et al. 2018, Journal of Economic Entomology 111, 2652-2659
206	Nesidiocoris tenuis	Hemiptera	Miridae	-0.0171	0.0016	>0.999	7	-0.0195	0.0014	>0.999	7	-0.010	-0.008	Mirhosseini et al. 2018, Environmental Entomology 47, 467-476
207	Aleurodicus dugesii	Hemiptera	Aleyrodidae	0.0423	NA	0.997	4	0.0357	0.0024	0.999	4	0.171	0.106	Schoeller & Redak 2018, Environmental Entomology 47, 1586-1595
208	Pachycrepoideus vindemmiae	Hymenoptera	Pteromalidae	NA	NA	NA	NA	-0.0137	0.0028	>0.999	8	NA	0.054	Wang et al. 2018, Environmental Entomology 47, 764-772
209	Pachycrepoideus vindemmiae	Hymenoptera	Pteromalidae	NA	NA	NA	NA	0.0081	0.0030	>0.999	7	NA	0.039	Wang et al. 2018, Environmental Entomology 47, 764-772
210	Trichopria drosophilae	Hymenoptera	Diapriidae	NA	NA	NA	NA	-0.0350	0.0025	>0.999	4	NA	0.063	Wang et al. 2018, Environmental Entomology 47, 764-772
211	Lobesia botrana	Lepidoptera	Tortricidae	0.0497	0.0093	NA	2	NA	NA	NA	2	0.079	NA	Iltis et al. 2019, Journal of Insect Physiology 117, 103916
212	Graphosoma lineatum	Hemiptera	Pentatomidae	0.0224	0.0036	>0.999	4	0.0224	0.0034	>0.999	4	-0.018	-0.018	Lopatina & Gusev 2019, Entomological Review 99, 417-436
213	Graphosoma lineatum	Hemiptera	Pentatomidae	0.0033	0.0026	>0.999	5	0.0033	0.0025	>0.999	5	-0.007	-0.007	Lopatina & Gusev 2019, Entomological Review 99, 417-436
214	Graphosoma lineatum	Hemiptera	Pentatomidae	0.0310	0.0021	0.998	4	0.0310	0.0022	0.998	4	-0.018	-0.018	Lopatina & Gusev 2019, Entomological Review 99, 417-436
215	Graphosoma lineatum	Hemiptera	Pentatomidae	0.0059	0.0016	>0.999	5	0.0059	0.0016	>0.999	5	0.007	0.007	Lopatina & Gusev 2019, Entomological Review 99, 417-436
216	Diaphorencyrtus aligarhensis	Hymenoptera	Encyrtidae	NA	NA	NA	NA	-0.0185	0.0030	>0.999	6	NA	-0.012	Milosavljevi? et al. 2019, Journal of Economic Entomology 112, 1062-1072
217	Diaphorencyrtus aligarhensis	Hymenoptera	Encyrtidae	NA	NA	NA	NA	0.0496	0.0028	0.984	6	NA	-0.059	Milosavljevi? et al. 2019, Journal of Economic Entomology 112, 1062-1072
218	Ostrinia furnacalis	Lepidoptera	Crambidae	-0.0299	0.0017	0.999	6	NA	NA	NA	6	0.021	NA	Xia et al. 2019, Physiological Entomology 44, 209-214
219	Chrysodeixis chalcites	Lepidoptera	Noctuidae	NA	NA	NA	NA	0.0171	0.0037	>0.999	4	NA	-0.018	del Pino et al. 2020, Environmental Entomology 49, 777-788
220	Bicyclus anynana	Lepidoptera	Nymphalidae	-0.0038	0.0047	>0.999	3	NA	NA	NA	3	0.062	NA	Singh et al. 2020, Evolutionary Ecology 34, 713-734
221	Bicyclus anynana	Lepidoptera	Nymphalidae	-0.0056	0.0056	>0.999	3	NA	NA	NA	3	0.062	NA	Singh et al. 2020, Evolutionary Ecology 34, 713-734
222	Microplitis similis	Hymenoptera	Braconidae	0.0934	0.0042	0.992	6	0.0600	0.0030	0.995	6	-0.019	-0.023	Yi et al. 2020, Physiological Entomology 45, 95-102
223	Culex pipiens	Diptera	Culicidae	0.4551	0.0273	NA	2	NA	NA	NA	2	0.074	NA	Alcalay et al. 2018, Journal of Zoology 306, 268-278
224	Culex pipiens	Diptera	Culicidae	0.1081	0.0123	NA	2	NA	NA	NA	2	0.096	NA	Alcalay et al. 2018, Journal of Zoology 306, 268-278
225	Chilo suppressalis	Lepidoptera	Crambidae	0.1304	NA	0.994	4	NA	NA	NA	4	0.080	NA	Huang et al. 2018, Ecology and Evolution 8, 12694-12701
226	Chilo suppressalis	Lepidoptera	Crambidae	0.0206	NA	0.999	3	NA	NA	NA	3	0.064	NA	Huang et al. 2018, Ecology and Evolution 8, 12694-12701
227	Diadegma mollipla	Hymenoptera	Ichneumonidae	NA	NA	NA	NA	0.0068	0.0200	>0.999	4	NA	-0.001	Sithole et al. 2017, BioControl 62, 603-612
228	Hyphantria cunea	Lepidoptera	Erebidae	0.1988	0.0352	0.978	3	NA	NA	NA	3	0.046	NA	Jang et al. 2015, Entomologia Experimentalis et Applicata 154, 120-130
229	Hyphantria cunea	Lepidoptera	Erebidae	-0.0628	0.0297	0.986	3	NA	NA	NA	3	0.029	NA	Jang et al. 2015, Entomologia Experimentalis et Applicata 154, 120-130
230	Aedes aegypti	Diptera	Culicidae	NA	NA	NA	NA	0.0612	0.0097	0.999	4	NA	0.143	Farjana et al. 2012, Medical and Veterinary Entomology 26, 210-217
231	Aedes aegypti	Diptera	Culicidae	NA	NA	NA	NA	-0.1067	0.0103	0.995	4	NA	0.197	Farjana et al. 2012, Medical and Veterinary Entomology 26, 210-217
232	Aedes albopictus	Diptera	Culicidae	NA	NA	NA	NA	-0.0780	0.0103	0.975	4	NA	0.062	Farjana et al. 2012, Medical and Veterinary Entomology 26, 210-217
233	Aedes albopictus	Diptera	Culicidae	NA	NA	NA	NA	0.0097	0.0109	0.984	4	NA	0.129	Farjana et al. 2012, Medical and Veterinary Entomology 26, 210-217
234	Bicyclus anynana	Lepidoptera	Nymphalidae	-0.0173	0.0024	NA	2	NA	NA	NA	2	0.060	NA	Steigenga & Fischer 2009, Journal of Thermal Biology 34, 244-249
235	Riptortus pedestris	Hemiptera	Alydidae	0.0068	0.0008	>0.999	7	0.0005	0.0006	>0.999	7	-0.003	-0.004	Ahn et al. 2019, Applied Entomology and Zoology 54, 63-74
236	Callosobruchus chinensis	Coleoptera	Chrysomelidae	NA	NA	NA	NA	0.0198	0.0205	NA	2	NA	0.037	Terada et al. 2019, Applied Entomology and Zoology 54, 459-464
237	Callosobruchus chinensis	Coleoptera	Chrysomelidae	NA	NA	NA	NA	0.0810	0.0187	NA	2	NA	0.041	Terada et al. 2019, Applied Entomology and Zoology 54, 459-464
238	Callosobruchus chinensis	Coleoptera	Chrysomelidae	NA	NA	NA	NA	0.0163	0.0163	NA	2	NA	0.046	Terada et al. 2019, Applied Entomology and Zoology 54, 459-464
239	Callosobruchus chinensis	Coleoptera	Chrysomelidae	NA	NA	NA	NA	-0.0399	0.0229	NA	2	NA	0.025	Terada et al. 2019, Applied Entomology and Zoology 54, 459-464
240	Callosobruchus chinensis	Coleoptera	Chrysomelidae	NA	NA	NA	NA	0.0570	0.0170	NA	2	NA	0.042	Terada et al. 2019, Applied Entomology and Zoology 54, 459-464
241	Callosobruchus chinensis	Coleoptera	Chrysomelidae	NA	NA	NA	NA	-0.0716	0.0178	NA	2	NA	0.041	Terada et al. 2019, Applied Entomology and Zoology 54, 459-464
242	Aethina tumida	Coleoptera	Nitidulidae	NA	NA	NA	NA	-0.0330	0.0022	>0.999	5	NA	-0.075	Noor-ul-Ane & Jung 2020, Journal of Apicultural Research 59, 807-816
243	Brachymeria lasus	Hymenoptera	Chalcididae	NA	NA	NA	NA	0.0377	0.0068	0.998	5	NA	0.041	Tian et al. 2020, Journal of Forest Research 32, 401-407
