# Supplementary material for: Limited sex differences in plastic responses suggest evolutionary conservatism of thermal reaction norms: A meta‐analysis in insects
Source: Evol Lett. 2022 Nov 2;6(6):394–411. doi: 10.1002/evl3.299 (PMC9783480; doi:10.1002/evl3.299)
Supplement: Supplementary file 5 — Table S2. Sexual development time dimorphism (SDTD) in major insect orders, based on the species examined. [file EVL3-6-394-s007.pdf]

Supplementary Material

Table S2. Sexual development time dimorphism (SDTD) in major insect orders, based on the species examined. The percentages refer to the fraction of species with male-biased SDTD, separately for larval and total development (not shown for clades with less than seven data sets available). Figures in the parentheses refer to counts of individual species with male- and female-biased SDTD. Note that some species are represented by more than one data set.

**Insect order**	**Percentage of species with male-biased SDTD**	
	**Larval (m/f)**	**Total (m/f)**
Lepidoptera	10% (6/57)	37% (14/23)*
Coleoptera	63% (5/3)	41% (9/13)
Diptera	9% (2/20)*	33% (10/20)
Hymenoptera	20% (2/8)	15% (8/46)
Hemiptera	52% (17/16)	52% (17/16)
Orthoptera	0% (0/9)	0% (0/9)
**All species**	**22% (34/123)***	**31% (61/138)***

* no sex difference in one data set (i.e., SDTD = 0)
