# Supplementary material for: Limited sex differences in plastic responses suggest evolutionary conservatism of thermal reaction norms: A meta‐analysis in insects
Source: Evol Lett. 2022 Nov 2;6(6):394–411. doi: 10.1002/evl3.299 (PMC9783480; doi:10.1002/evl3.299)
Supplement: Supplementary file 6 — Table S3. Sources of original data and variables derived from these data: ln‐slope = logarithmically transformed RMA regression slope of male development time on female development time, gradient length = ratio of maximum and minimum development time. [file EVL3-6-394-s001.pdf]

**Supplementary Material**

**Table S3.** Sources of original data and variables derived from these data: ln-slope = logarithmically transformed RMA regression slope of male development time on female development time, gradient length = ratio of maximum and minimum development time.

## a) Sex differences in temperature-induced plasticity

| Species | Order | Ln-slope | Gradient length | Source |
|---|---|---|---|---|
| Aedes aegypti | Diptera | 0.023 | 2.165 | Farjana et al. 2012, Medical and Veterinary Entomology 26, 210-217 |
| Aedes albopictus | Diptera | 0.035 | 1.980 | Farjana et al. 2012, Medical and Veterinary Entomology 26, 210-217 |
| Aphidius colemani | Hymenoptera | 0.006 | 5.069 | Zamani et al. 2007, Environmental Entomology 36, 263-271 |
| Aphidius matricariae | Hymenoptera | 0.001 | 4.797 | Zamani et al. 2007, Environmental Entomology 36, 263-271 |
| Bicyclus anynana | Lepidoptera | 0.006 | 2.206 | Fischer et al. 2003, Ecology 84, 3138-3147 |
| Chaetophthalmus dorsalis | Diptera | 0.050 | 1.740 | Walker 2011, Australian Journal of Entomology 50, 309-318 |
| Chilo suppressalis | Lepidoptera | 0.131 | 1.418 | Huang et al. 2018, Ecology and Evolution 8, 12694-12701 |
| Crocidosema plebejana | Lepidoptera | 0.009 | 7.116 | Hamilton & Zalucki 1991, Australian Journal of Zoology 39, 191-200 |
| Dicyphus hesperus | Hemiptera | 0.036 | 3.335 | Gillespie et al. 2004, Canadian Entomologist 136, 675-683 |
| Diglyphus isaea | Hymenoptera | 0.031 | 3.538 | Bazzocchi et al. 2003, Biological Control 26, 74-82 |
| Epiphyas postvittana | Lepidoptera | 0.014 | 1.351 | Mo et al. 2006, Journal of Economic Entomology 99, 1321-1326 |
| Hermetia illucens | Diptera | 0.118 | 1.115 | Tomberlin et al. 2009, Environmental Entomology 38, 930-934 |
| Hyphantria cunea | Lepidoptera | 0.068 | 1.119 | Jang et al. 2015, Entomologia Experimentalis et Applicata 154, 120-130 |
| Leptinotarsa decemlineata | Coleoptera | 0.005 | 3.005 | Lyytinen et al. 2008, Entomologia Experimentalis et Applicata 127, 157-167 |
| Leptomastix epona | Hymenoptera | 0.104 | 2.080 | Karamaouna & Copland 2009, BioControl 54, 65-76 |
| Lobesia botrana | Lepidoptera | 0.050 | 1.429 | Iltis et al. 2019, Journal of Insect Physiology 117, 103916 |
| Lycaena tityrus | Lepidoptera | 0.092 | 1.533 | Fischer & Fiedler 2000, Oikos 90, 372-380 |
| Lymantria dispar | Lepidoptera | 0.930 | 1.402 | Lindroth et al. 1997, Physiological Entomology 22, 55-64;<br>Thompson et al. 2017, Physiological Entomology 42, 181-190 |
| Lysiphlebia mirzai | Hymenoptera | 0.022 | 3.545 | Liu & Tsai 2002, Environmental Entomology 31, 418-424 |
| Merophyas divulsana | Lepidoptera | 0.048 | 5.776 | Allsopp et al. 1983, Journal of Australian Entomological Society 22, 287-291 |
| Omocestus viridulus | Orthoptera | 0.098 | 2.678 | Willott & Hassall 1998, Functional Ecology 12, 232-241 |
| Orius naivashae | Hemiptera | 0.023 | 5.078 | Bonte et al. 2012, Environmental Entomology 41, 989-996 |
| Orius thripoborus | Hemiptera | 0.008 | 3.361 | Bonte et al. 2012, Environmental Entomology 41, 989-996 |

| | | | | |
|---|---|---|---|---|
| Pachycrepoideus vindemiae | Hymenoptera | 0.003 | 15.293 | Wang et al. 2018, Environmental Entomology 47, 764-772 |
| Phthorimaea operculella | Lepidoptera | 0.009 | 4.435 | Golizadeh & Zalucki 2012, Insect Science 19, 609-620 |
| Piezodorus guildinii | Hemiptera | 0.032 | 1.710 | Zerbino et al. 2013, Florida Entomologist 96, 572-582 |
| Plutella xylostella | Lepidoptera | 0.008 | 2.106 | Atwal 1955, Australian Journal of Zoology 3, 185-221 |
| Podisus maculiventris | Hemiptera | 0.013 | 2.371 | Legaspi & Legaspi 2005, Environmental Entomology 34, 990-998 |
| Scathophaga stercoraria | Diptera | 0.011 | 2.717 | Blanckenhorn 1997, Oecologia 111, 318-324; Blanckenhorn & Henseler 2005, Entomologia Experimentalis et Applicata 116, 159-165 |
| Trichogrammatoidea lutea | Hymenoptera | 0.003 | 2.841 | Mawela et al. 2013, Biological Control 64, 211-216 |

## b) Sex differences in diet-induced plasticity

| Species | Order | Ln-slope | Gradient length | Source |
|---|---|---|---|---|
| Aedes aegypti | Diptera | 0.159 | 1.449 | Farjana et al. 2012, Medical and Veterinary Entomology 26, 210-217 |
| Aedes albopictus | Diptera | 0.174 | 1.455 | Farjana et al. 2012, Medical and Veterinary Entomology 26, 210-217 |
| Aphidius colemani | Hymenoptera | 0.578 | 1.050 | Zamani et al. 2007, Environmental Entomology 36, 263-271 |
| Aphidius matricariae | Hymenoptera | 0.135 | 1.049 | Zamani et al. 2007, Environmental Entomology 36, 263-271 |
| Bicyclus anynana | Lepidoptera | 0.070 | 1.036 | Bauerfeind & Fischer 2005, Oikos 111, 514-524; Bauerfeind & Fischer 2009, Entomologia Experimentalis et Applicata 130, 229-237 |
| Chaetophthalmus dorsalis | Diptera | 0.045 | 1.261 | Walker 2011, Australian Journal of Entomology 50, 309-318 |
| Chilo suppressalis | Lepidoptera | 0.256 | 1.655 | Huang et al. 2018, Ecology and Evolution 8, 12694-12701 |
| Crocidosema plebejana | Lepidoptera | 0.088 | 1.348 | Hamilton & Zalucki 1993, Entomologia Experimentalis et Applicata 66, 199-205 |
| Dicyphus hesperus | Hemiptera | 0.106 | 1.086 | Gillespie & McGregor 2000, Ecological Entomology 25, 380-386 |
| Diglyphus isaea | Hymenoptera | 0.451 | 1.038 | Bazzocchi et al. 2003, Biological Control 26, 74-82 |
| Epiphyas postvittana | Lepidoptera | 0.007 | 1.290 | Mo et al. 2006, Journal of Economic Entomology 99, 1321-1326; Rizvi et al. 2016, Insect Science 23, 313-325 |
| Hermetia illucens | Diptera | 0.330 | 1.102 | Myers et al. 2008, Environmental Entomology 37, 11-15 |
| Hyphantria cunea | Lepidoptera | 0.130 | 1.033 | Jang et al. 2015, Entomologia Experimentalis et Applicata 154, 120-130 |
| Leptinotarsa decemlineata | Coleoptera | 0.753 | 1.049 | Lyytinen et al. 2008, Entomologia Experimentalis et Applicata 127, 157–167 |
| Leptomastix epona | Hymenoptera | 0.724 | 1.258 | Karamaouna & Copland 2000, Entomologia Experimentalis et Applicata 96, 149-158 |
| Lobesia botrana | Lepidoptera | 0.167 | 1.423 | Thiery & Moreau 2005, Oecologia 143, 548-557 |
| Lycaena tityrus | Lepidoptera | 0.105 | 1.091 | Fischer & Fiedler 2000, Oecologia 124, 235-241 |
| Lymantria dispar | Lepidoptera | 0.140 | 1.269 | Joseph & Kelsey 1994, Environmental Entomology 23, 396-405; Lazarević et al. 2002, Entomologia Experimentalis et Applicata 102, 75-86; |

| | | | | |
|---|---|---|---|---|
| | | | | Barbosa et al. 1983, Environmental Entomology 12, 1858-1862;<br>Lance et al. 1986, Ecology 67, 1650-1654;<br>Barbosa et al. 1986, Ecological Entomology 11, 1-6;<br>Stoyenoff et al. 1994, Oecologia 97, 143-157;<br>Kruse & Raffa 1997, Environmental Entomology 26, 1155-1166;<br>Strom & Hain 1996, Environmental Entomology 25, 603-610;<br>Lindroth et al. 1997, Physiological Entomology 22, 55-64 |
| Lysiphlebia mirzai | Hymenoptera | 0.716 | 1.169 | Pandey & Singh 1999, Entomologia Experimentalis et Applicata 90, 61-67 |
| Merophyas divulsana | Lepidoptera | 0.053 | 1.695 | Allsopp et al. 1983, Journal of the Australian Entomological Society 22, 287-291 |
| Omocestus viridulus | Orthoptera | 0.167 | 1.139 | Berner et al. 2005, Oikos 111, 525-533 |
| Orius naivashae | Hemiptera | 0.021 | 1.329 | Bonte et al. 2012, Journal of Insect Science 12:1 |
| Orius thripoborus | Hemiptera | 0.067 | 1.232 | Bonte et al. 2012, Journal of Insect Science 12:1 |
| Pachycrepoideus vindemiae | Hymenoptera | 0.592 | 1.135 | Wang & Messing 2004, Biological Control 31, 227-236 |
| Phthorimaea operculella | Lepidoptera | 0.651 | 1.282 | Horgan et al. 2007, Entomologia Experimentalis et Applicata 125, 249-258;<br>Horgan et al. 2012, Neotropical Entomology 41, 333-340 |
| Piezodorus guildinii | Hemiptera | 0.204 | 1.384 | Panizzi 1992, Entomologia Experimentalis et Applicata 63, 221-228 |
| Plutella xylostella | Lepidoptera | 0.087 | 1.208 | Sarfraz et al. 2007, Journal of Economic Entomology 100, 215-224;<br>Barker et al. 2006, Entomologia Experimentalis et Applicata 122, 17-26;<br>Sarfraz et al. 2010, Canadian Entomologist 142, 24-35;<br>Atwal 1955, Australian Journal of Zoology 3, 185-221 |
| Podisus maculiventris | Hemiptera | 0.043 | 2.559 | Mahdian et al. 2006, BioControl 51, 725-739 |
| Scathophaga stercoraria | Diptera | 0.097 | 1.152 | Blanckenhorn & Heyland 2004, Evolutionary Ecology 18, 385-402;<br>Amano 1983, Japanese Journal of Sanitary Zoology 34, 165-175 |
| Trichogrammatoidea lutea | Hymenoptera | 0.018 | 1.182 | Mawela et al. 2013, Biological Control 64, 211-216 |
